# Unveiling the Pulmonary Toxicity of Polystyrene Nanoplastics: A Hierarchical Oxidative Stress Mechanism Driving Acute–Subacute Lung Injury

**DOI:** 10.34133/research.0995

**Published:** 2025-11-24

**Authors:** Xianyi Tang, Lu Lu, Yuqing Sun, Aiyun Li, Wanzhen Su, Jimin Cao, Xiao Zhang, Xi Liu, Yanlin Feng

**Affiliations:** ^1^ Department of Cardiology, the First Hospital of Shanxi Medical University, and Key Laboratory of Cellular Physiology at Shanxi Medical University, Ministry of Education, Taiyuan 030001, China.; ^2^ Institute of NBC Defence, PLA Army, Beijing 102205, China.; ^3^ Second Clinical Medical College, School of Pharmacy and Key Laboratory of Cellular Physiology, Shanxi Medical University, Taiyuan 030001, China.; ^4^ Medical Innovation Research Division, Chinese PLA General Hospital, Beijing 100048, China.

## Abstract

The ubiquitous contamination of airborne nanoplastics (<100 nm), particularly polystyrene nanoplastics (PS NPs), has emerged as critical determinant regarding respiratory health. As inhalation exposure represents a primary route of human contact, the pulmonary toxicological profiles of PS NPs remained poorly characterized, with underlying mechanisms lacking. In this study, we systematically investigated the pulmonary toxicological mechanisms of PS NPs for inducing acute–subacute lung injury. PS NPs markedly reduced cell viability of lung epithelial cells (BEAS-2B cells) and macrophages (RAW 264.7 cells) in a dose-dependent manner. Further investigations revealed that PS NPs induced a hierarchical oxidative stress paradigm that redox imbalance triggered by reactive oxygen species (ROS) burst led to the cascade activation of compensatory nuclear factor erythroid 2-related factor 2 (Nrf2)/heme oxygenase-1 (HO-1) pathway, up-regulation of pro-inflammatory cytokines, mitochondrial dysfunction, and apoptosis. Furthermore, the in vivo toxicities of PS NPs were validated by the acute and subacute pulmonary injury on rodent models, which was characterized by obvious inflammatory cell infiltration and pulmonary fibrosis. These results indicated that hierarchical oxidative stress mediated PS NPs-induced acute–subacute lung injury. This also raised a warning that susceptible individuals with long-term plastic exposure should receive regular pulmonary monitoring.

## Introduction

The widespread plastic products have led to substantial environmental contamination, posing a global public health challenge. By 2050, the cumulative volume of plastic waste worldwide is forecast to hit 33 billion tons, with only 27% estimated to be recycled [1–3]. The degraded plastic product-derived micro/nanoparticles have been identified as potential exposure hazards through ingestion, inhalation, and dermal contact [4–7]. Of particular concern is their pervasive presence in human tissues (e.g., placentas, brains, lung, liver, and intestines) and body fluids (e.g., breast milk and blood) [8–13]. Moreover, nanoplastics are emphasized as a critical concern not only due to their deeper penetration in tissues but also for their larger specific surface area [12,14,15]. Among the various toxicities caused by nanoplastics, pulmonary toxicity has garnered increasing attention [11,16,17]. Nanoplastics are capable of global transport through atmospheric circulation owing to their low density [18,19]. A computational fluid-particle dynamics (CFPD) model developed by researchers at the University of Technology Sydney has demonstrated that smaller nanoplastics are more likely to evade initial deposition mechanisms and migrate to deeper airway regions [20].

Polystyrene nanoplastics (PS NPs) have been the most used plastic type in food packaging and biomedical fields due to its excellent mechanical properties, transparency, and low cost [21,22]. Consequently, the toxicity of PS NPs has gained widespread attention. Recent in vitro evidence reported that smaller PS NPs with a diameter of 25 nm exhibit much higher cell uptake in alveolar epithelial cells (such as A549 cells) than their larger counterparts (70 nm), leading to the dysregulation of cell cycle and activation of caspase pathway [23]. Several experimental studies have revealed that PS NPs can induce oxidative stress, inflammation, and epithelial barrier destruction on human alveolar epithelial cells as well as in intratracheally exposure or oral–nasal inhalation rodent models of chronic obstructive pulmonary diseases (COPDs) [24–26]. However, the pulmonary toxicity and mechanisms of PS NPs on acute–subacute lung injury have not been discovered.

Hierarchical oxidative stress paradigm has been developed to explain the role of oxidative stress in mediating the biological effects of nanoparticles [27]. This 3-tier model posits that low levels of oxidative stress induce protective effects to avoid more damaging effects at higher oxidative stress levels. At tier 1, the transcription factor nuclear factor erythroid 2-related factor 2 (Nrf2) increases the expression of phase II antioxidant enzymes, such as heme oxygenase-1 (HO-1), to restore cellular redox homeostasis and achieve the protective effects. When excessive production of reactive oxygen species (ROS) exceeds the antioxidant scavenging ability, the increased stress can trigger the redox-sensitive mitogen-activated protein kinase (MAPK) and nuclear factor κB (NF-κB) cascades, which are responsible for the expression of pro-inflammatory cytokines and adhesion molecules [28], many of which are involved in the inflammatory process of the lung (tier 2). Upon failure of pro-inflammatory responses, all the defense systems are overwhelmed and sustained oxidative stress progresses to tier 3. Mitochondrial dysfunction and compromised electron transfer in this stage trigger apoptotic cell death [29,30]. Taken together, the hierarchical cellular oxidative stress model may provide a mechanistic platform to understand the pulmonary toxicity and mechanisms of PS NPs in acute–subacute lung injury.

**Fig. 1.**
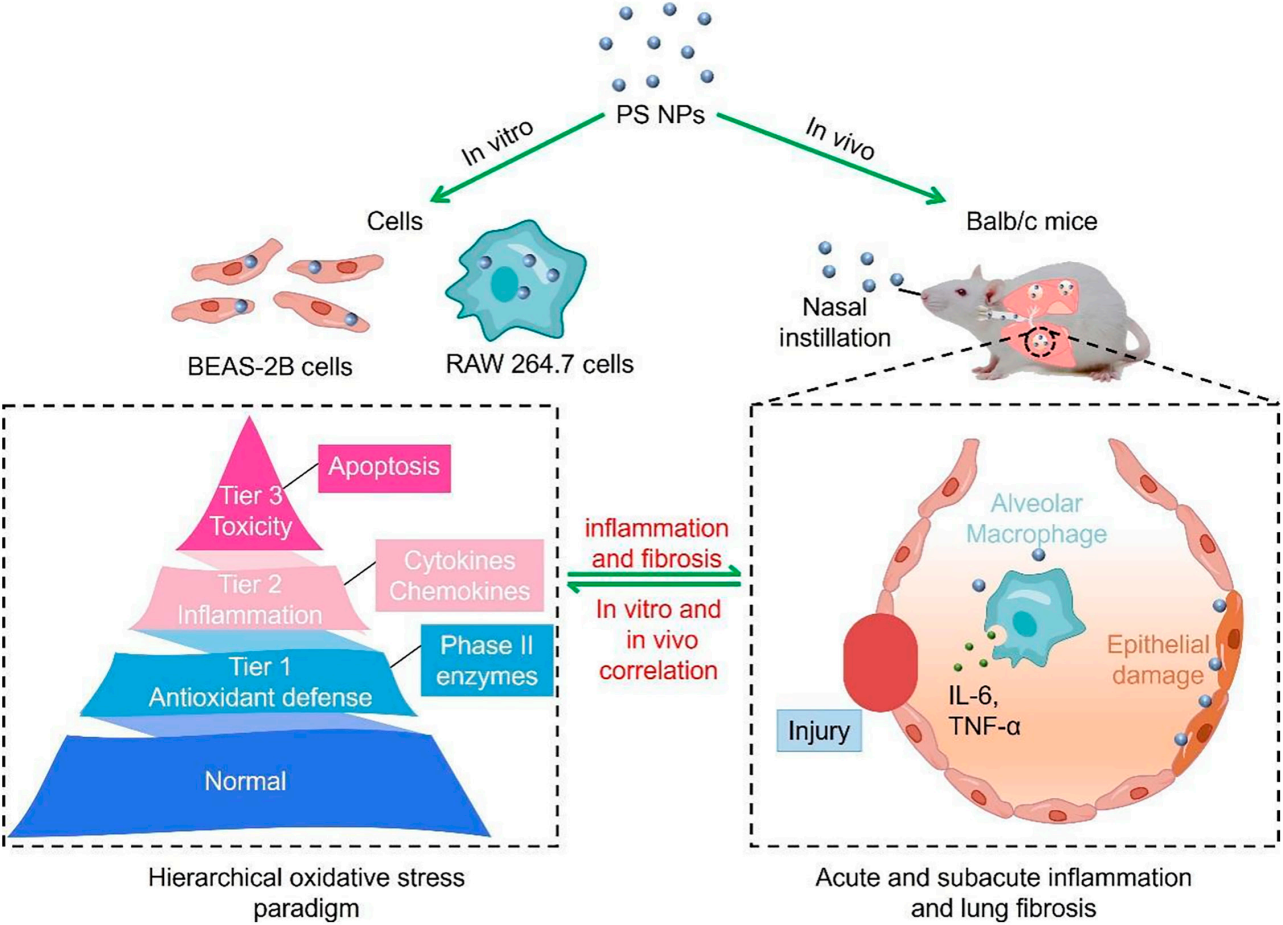
Schematic illustration displaying that hierarchical oxidative stress drove the in vitro and in vivo pulmonary toxicological effects of PS NPs.

Herein, PS NPs (42.1 ± 5.4 nm) were purchased to systematically evaluate the lung toxicology effects of plastic products from in vitro to in vivo models. First, the toxicological effects and mechanisms of PS NPs were studied on human bronchial epithelial cells (BEAS-2B) and murine monocyte–macrophage leukemia cells (RAW 264.7). The excess accumulation of ROS levels, up-regulated expression of Nrf2 and HO-1, increased expression of pro-inflammatory cytokines, obvious mitochondrial dysfunction, and apoptosis were observed on both BEAS-2B and RAW 264.7 cells, indicating that the hierarchical oxidative stress drove the toxic outcomes of PS NPs. Then, the toxicological effects of PS NPs were investigated on the acute and subacute pulmonary injury on rodent models. Representative inflammatory cell infiltration, up-regulated expression of pro-inflammatory cytokines, and pulmonary fibrosis were observed on Balb/c mice exposed to PS NPs (Fig. 1). Therefore, this study revealed that the hierarchical oxidative stress mechanism drove PS NPs-induced acute–subacute lung injury.

## Results and Discussion

### Characterization of PS NPs

**Fig. 2.**
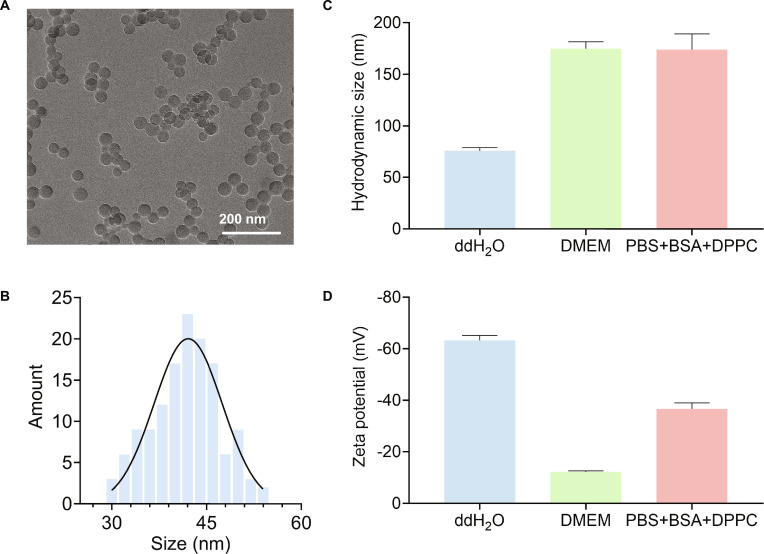
Characterization of PS NPs. (A) Representative TEM images of PS NPs. (B) Size distribution of PS NPs. (C and D) Hydrodynamic sizes and zeta potential of PS NPs in ddH_2_O, DMEM, and surrogate lung lining fluid.

Transmission electron microscopy (TEM) analysis showed that PS NPs possessed a spherical morphology with smooth surface and a uniform diameter of 42.1 ± 5.4 nm (Fig. 2A and B). Figure 2C reveals that these PS NPs demonstrated effective dispersion in aqueous media, including double-distilled water (ddH_2_O), Dulbecco’s modified Eagle’s medium (DMEM), and the surrogate lung lining fluid [phosphate-buffered saline (PBS) supplemented with 0.6 mg/ml bovine serum albumin (BSA) and 0.01 mg/ml dipalmitoylphosphatidylcholine (DPPC)]. Dynamic light scattering (DLS) measurements showed that the hydrodynamic diameter of PS NPs was 75.8 ± 3.07 nm in water, which increased to 174.8 ± 6.68 nm in DMEM and 173.8 ± 15.26 nm in the surrogate lung lining fluid (Fig. 2C). The increased hydrodynamic size may be ascribed to the formation of a protein corona [31–33]. Next, zeta potential measurements further confirmed the colloidal stability of PS NPs, with surface charges shifting from −63.3 ± 1.92 mV in water to −12.2 ± 0.40 mV in DMEM and −36.7 ± 2.30 mV in the surrogate lung lining fluid (Fig. 2D). This stability can be attributed to interparticle electrostatic repulsion forces, which were effective in preventing aggregation across these different media [34]. Moreover, the green fluorescent PS NPs (_GF_PS NPs) exhibited comparable physicochemical characteristics to their nonfluorescent counterparts (Fig. S1). Collectively, these results demonstrate the robust stability and homogeneous dispersibility of PS NPs in various media.

### PS NPs induced the hierarchical oxidative stress responses in BEAS-2B cells

**Fig. 3.**
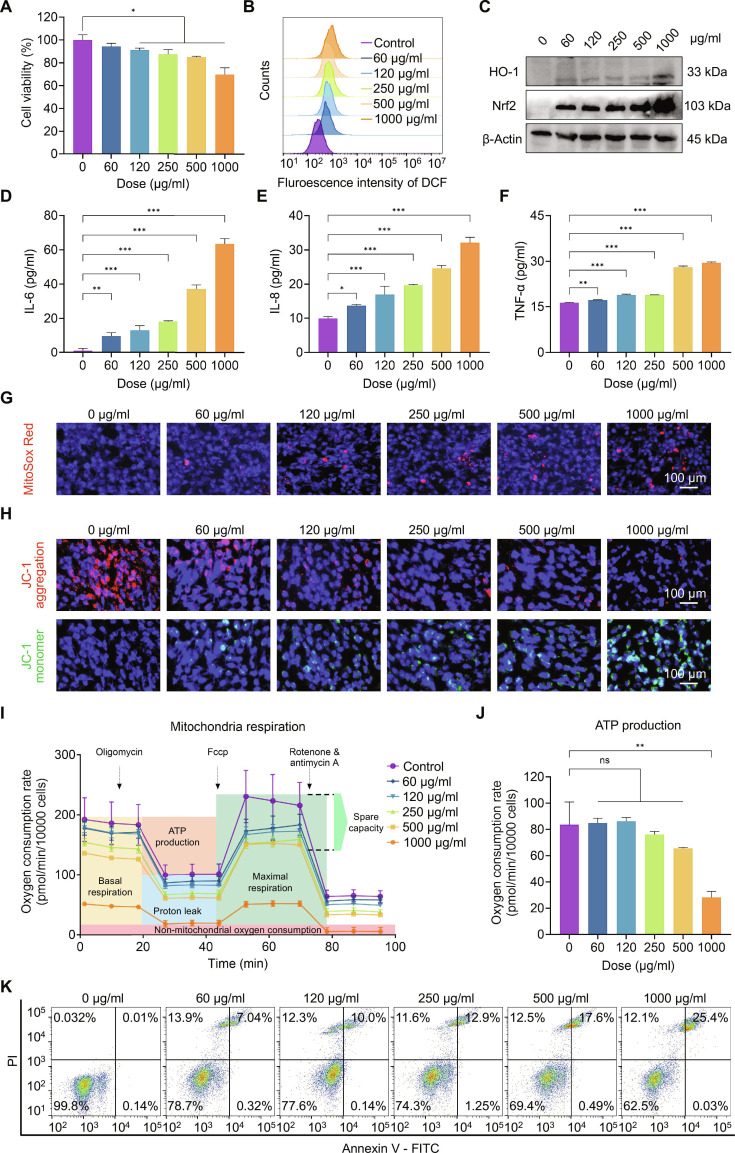
PS NPs induced the hierarchical oxidative stress responses in BEAS-2B cells. (A) CCK-8 assay indicating the cytotoxicity of PS NPs on BEAS-2B cells. (B) Cellular ROS level based on flow cytometry assay. (C) Western blot images showing the expression of cellular Nrf2 and HO-1. (D to F) ELISA assay assessing the secretion of IL-6, IL-8, and TNF-α in BEAS-2B cells. (G and H) MitoSox and JC-1 staining showing the generation of mitochondrial superoxide and depolarization of mitochondrial membrane. (I) OCR measured by Seahorse MitoStress assay in BEAS-2B cells. (J) ATP production of PS NPs-treated BEAS-2B cells. (K) Flow cytometric analysis showing apoptotic BEAS-2B cells using Annexin V–FITC/PI assay. ns is not significant, *P < 0.05, **P < 0.01, ***P < 0.001 versus the 0 μg/ml group.

Considering that inhalation is the predominant exposure route for PS NPs, the human bronchial epithelial cells (BEAS-2B) were employed as an in vitro pulmonary toxicity model, which provide a human-relevant airway epithelium model that directly reflects the initial contact site of inhaled nanoparticles [35–39]. First, the cellular internalization of _GF_PS NPs by BEAS-2B cells was investigated by fluorescence microscopy. Fluorescent images demonstrated a pronounced perinuclear accumulation of _GF_PS NPs following 24-h co-incubation, confirming their successful cellular internalization (Fig. S2). Then, the cytotoxicity of PS NPs was measured by the Cell Counting Kit-8 (CCK-8) assay. Figure 3A exhibits a concentration-dependent toxicity effect of PS NPs on BEAS-2B cells after 24-h co-incubation. Notably, the cell viability of BEAS-2B cells was decreased sharply to 69.7 ± 5.95% when they were co-incubated with 1,000 μg/ml PS NPs. Meanwhile, the live/dead cell staining assay (Fig. S3) further validated this concentration–effect relationship. Next, cellular ROS levels in BEAS-2B cells treated with PS NPs were evaluated using 2′,7′-dichlorodihydrofluorescein diacetate (DCFH-DA) assay. Once inside the cells, nonfluorescent DCFH-DA is deacetylated by intracellular esterases to form DCFH, which is subsequently oxidized by cellular ROS to generate the highly fluorescent compound 2′,7′-dichlorofluorescein (DCF). Both flow cytometry analysis (Fig. 3B) and fluorescent images (Fig. S4) revealed that green fluorescent intensity of DCF was enhanced gradually by the increased concentration of PS NPs, which indicated the initiation of oxidative stress inside BEAS-2B cells.

According to typical hierarchical oxidative stress paradigm [40], Nrf2, the critical regulator of antioxidant stress response in tier 1, could be up-regulated to mediate the expression of phase II antioxidant enzymes (HO-1) to restore cellular redox homeostasis [41]. In our experiment, we detected that the protein levels of Nrf2 and HO-1 were up-regulated when the concentration of PS NPs increased from 60 to 1,000 μg/ml (Fig. 3C and Figs. S5 and S6). Subsequently, excess ROS further activated downstream MAPK and NF-κB pathways to trigger a robust inflammatory response mediated by various cytokines and chemokines. Enzyme-linked immunosorbent assay (ELISA) quantification revealed the dose-dependent elevation of pro-inflammatory mediators, including interleukin-6 (IL-6), IL-8, and tumor necrosis factor-α (TNF-α) in BEAS-2B cells exposed to PS NPs (Fig. 3D to F and Fig. S7).

Furthermore, the sustained oxidative stress progressed to trigger apoptosis through mitochondrial dysfunction in tier 3. Therefore, mitochondrial superoxide production and mitochondrial membrane depolarization of PS NPs-treated BEAS-2B cells were detected by MitoSox Red and JC-1 staining assay. Figure 3G and Fig. S8 illustrate that red fluorescence intensity of MitoSox was progressively increased with higher PS NPs concentrations, indicating that PS NPs resulted in the accumulation of mitochondrial superoxide in BEAS-2B cells. Meanwhile, the depolarization of mitochondrial membrane potential (Δψ_m_) serves as a hallmark event in early apoptotic activity. In healthy cells, the higher Δψ_m_ causes JC-1 to appear in cells in the form of aggregates and emits red fluorescence. When Δψ_m_ decreases, JC-1 shifts to its monomeric form and shows green fluorescence. Therefore, a drop in Δψ_m_ could be measured by the ratio of green/red fluorescence of JC-1 [42]. As shown in Fig. 3H and Fig. S9, untreated BEAS-2B cells mainly exhibited predominant red fluorescence from JC-1 aggregates, indicative of a high Δψ_m_. On the contrary, green fluorescence from JC-1 monomer appeared in PS NPs-treated BEAS-2B cells and the intensity of green fluorescence was increased when the concentration of PS NPs was raised from 60 to 1,000 μg/ml, indicating that PS NPs reduced Δψ_m_ in a dose-dependent manner. In fact, Δψ_m_ is crucial to maintain the physiological function of the respiratory chain. Thus, the real-time oxygen consumption rate (OCR), an indicator of mitochondrial respiration, was monitored by XFe seahorse systems. Figure 3I reveals a dose-dependent inhibition effect of PS NPs in the mitochondrial stress profiles in BEAS-2B cells. Basal respiration was reduced by 41.0 ± 10.44% when the concentration of PS NPs reached 1,000 μg/ml, accompanied by remarkable reductions in proton leak (12.8 ± 2.07%) and maximal respiratory capacity (46.7 ± 10.92%) (Fig. S10). In contrast, OCR linked to adenosine triphosphate (ATP) production showed a transient increase at lower concentrations attributed to mild uncoupling, followed by a graded decline as the electron transport chain became impaired (Fig. 3J). These observations illustrated a dynamic transition from a compensatory state to decompensated state in the process of ATP production. Taken together, PS NPs induced mitochondrial dysfunction in BEAS-2B cells. Finally, apoptosis represents the terminal outcome of oxidative stress injury, which was analyzed by Annexin V–fluorescein isothiocyanate (FITC)/propidium iodide (PI) double staining. Figure 3K displayed that PS NPs induced apoptosis in a dose-dependent manner, confirming the activation of cell death pathways. Bcl-2, a pivotal anti-apoptotic protein that preserves the integrity of the mitochondrial outer membrane [43,44], was further examined to substantiate the mechanistic connection between mitochondrial damage and cell apoptosis. As shown in Fig. S11, Bcl-2 protein expression decreased progressively with increasing PS NP concentrations, corroborating the activation of the mitochondrial-dependent apoptotic pathway. All considered, these findings indicate that PS NPs induced hierarchical oxidative stress responses in BEAS-2B cells, evolving from adaptive antioxidant activation to oxidative damage, mitochondrial collapse, and apoptotic cell death. This stepwise progression provides a compelling mechanistic explanation for the observed cytotoxicity, underscoring hierarchical oxidative stress as the central pathway mediating PS NPs-induced pulmonary toxicity. These results are consistent with previous studies reporting that oxidative stress-mediated mitochondrial dysfunction constitutes a major mechanism of nanoplastic cytotoxicity [45]. The observed Bcl-2 down-regulation parallels the collapse of mitochondrial membrane potential and subsequent cytochrome c release reported in other polymeric nanoparticle systems [46–48].

### PS NPs induced the hierarchical oxidative stress responses in RAW 264.7 cells

**Fig. 4.**
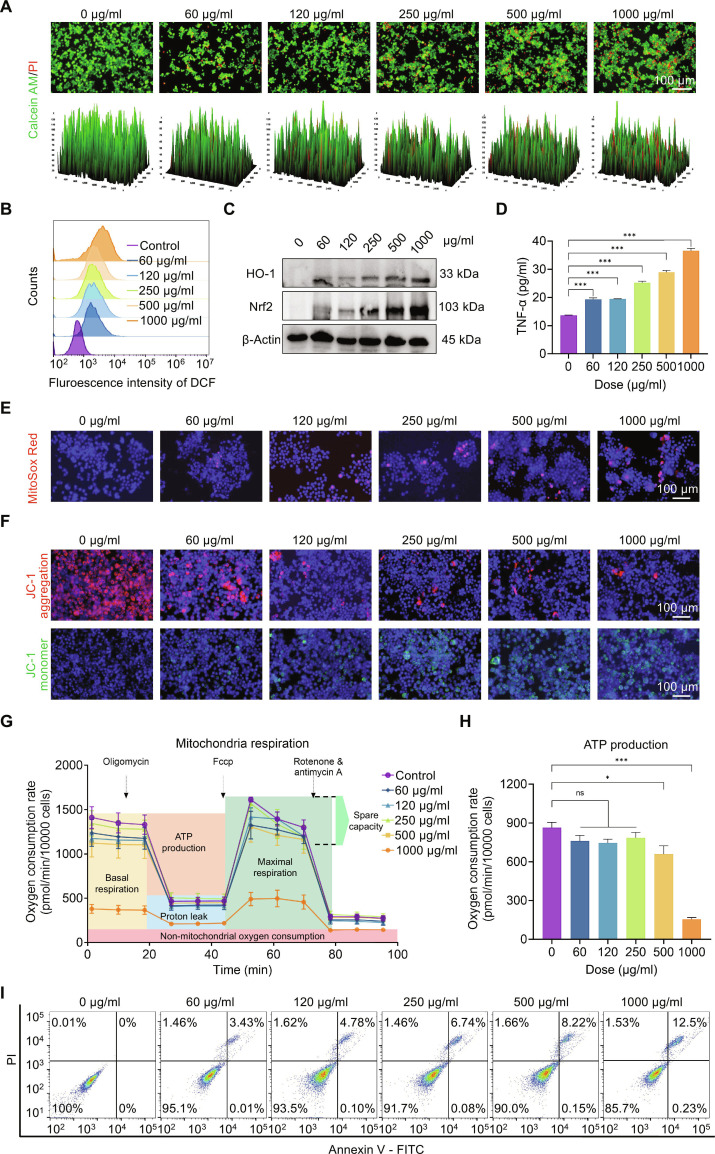
PS NPs induced hierarchical oxidative stress responses in RAW 264.7 cells. (A) Live/dead staining and corresponding 3D surface plots indicating the cell viability of RAW 264.7 cells following exposure to PS NPs. (B) ROS production in cells based on flow cytometry. (C) Western blot analysis of the expression levels of Nrf2 and HO-1. (D) ELISA assay assessing the production of TNF-α. (E and F) MitoSox and JC-1 staining showing mitochondrial superoxide generation and mitochondrial membrane depolarization. (G) OCR measured by Seahorse MitoStress assay in RAW 264.7 cells. (H) ATP production in PS NPs-treated RAW 264.7 cells. (I) Flow cytometric analysis showing apoptotic RAW 264.7 cells using Annexin V–FITC/PI assay. ns is not significant, *P < 0.05, ***P < 0.001 versus the 0 μg/ml group.

Given the pivotal contribution of alveolar macrophages to particulate clearance, we systematically evaluated the toxicological effect of PS NPs on RAW 264.7 murine macrophages. Fluorescence microscopy confirmed effective PS NPs endocytosis, with pronounced intracellular accumulation in perinuclear regions (Fig. S12). Quantitative viability assessment via CCK-8 assay demonstrated concentration-dependent cytotoxicity, showing 29.6 ± 0.18% reduction in cell activity at 1,000 μg/ml exposure of PS NPs compared to untreated control (Fig. S13). Similarly, the live/dead cell staining and the corresponding 3-dimensional (3D) surface plots revealed a decrease in the number of viable (green) RAW 264.7 cells and an increase in dead (red) cells with rising PS NPs concentrations (Fig. 4A). Furthermore, flow cytometry analysis and fluorescence imaging confirmed enhanced generation of intracellular ROS levels in PS NPs-treated RAW 264.7 cells (Fig. 4B and Fig. S14). Then, we also investigated the levels of Nrf2 and HO-1 proteins and observed that they were up-regulated with the rising concentration of PS NPs (Fig. 4C and Figs. S15 and S16). Similar phenomenon was observed in the inflammatory expression of IL-6 and TNF-α (Fig. 4D and Fig. S17A and B). Next, the inductive role of PS NPs on mitochondrial dysfunction in RAW 264.7 cells was further validated via MitoSox (Fig. 4E and Fig. S18) and JC-1 (Fig. 4F and Fig. S19).

Seahorse real-time metabolic analysis revealed PS NPs-mediated OCR suppression in RAW 264.7 cells with cell type-specific variations (Fig. 4G and H). Notably, RAW 264.7 macrophages did not induce immediate inhibition of mitochondrial respiratory function at low-to-medium concentrations of PS NPs (60 to 250 μg/ml). Instead, it triggered adaptive enhancement in basal respiration, proton leak, and maximal respiratory capacity (Fig. S20). We speculated that this unique response pattern may be attributed to the specialized nature of macrophages: The cells alleviated oxidative stress through mitochondria mild uncoupling and up-regulated energy metabolism to meet the demands of particle internalization and pro-inflammatory activation. However, this compensatory phase was limited. When the exposure concentration further increased (≥500 μg/ml), the compensatory mechanisms were overwhelmed, and mitochondria exhibited typical dose-dependent functional suppression, which was similar to the pattern observed in BEAS-2B cells.

Once mitochondrial compensatory competence was overwhelmed, the intrinsic apoptotic pathway became activated [49–52]. Apoptosis analysis and the expression of Bcl-2 confirmed the involvement of mitochondria in cell death pathways (Fig. 4I and Fig. S21). The findings from RAW 264.7 macrophages demonstrated that PS NPs induced a hierarchical oxidative stress response identical to that observed in bronchial epithelial cells, yet with notable cell type-specific characteristics. Macrophages effectively internalized PS NPs, triggering a cascade of events including antioxidant up-regulation (Nrf2/HO-1), pro-inflammatory activation (IL-6 and TNF-α), and mitochondrial impairment. These results underscore the susceptibility of immune cells to nanoplastic toxicity and highlight how cellular function shapes distinct stress response patterns within the hierarchical oxidative stress framework. This macrophage-specific vulnerability is consistent with their physiological role as first-line phagocytic defenders, where excessive ROS generation and cytokine release can paradoxically lead to self-destructive oxidative injury. Such findings reflect the mechanism observed in particulate matter exposure models, in which macrophage overactivation propagates inflammatory damage to neighboring epithelial cells. Therefore, our data not only elucidate the cell-intrinsic oxidative stress response of macrophages but also provide a mechanistic basis for intercellular cross-talk driving inflammation and fibrosis in PS NP-exposed lungs.

### NAC attenuated PS NPs-induced ROS production and apoptosis

**Fig. 5.**
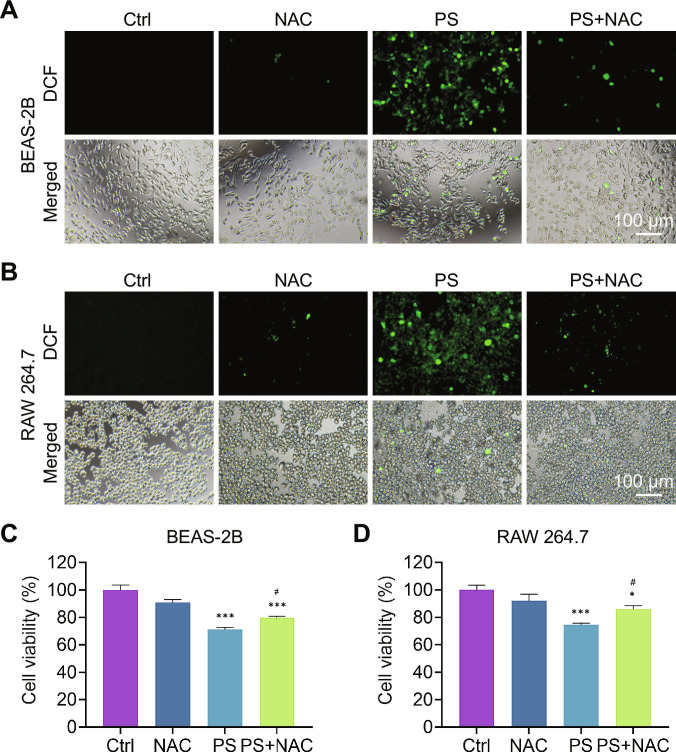
NAC attenuated PS NPs-induced ROS production and cytotoxicity. (A and B) Intracellular ROS level in BEAS-2B and RAW 264.7 cells based on DCF test. (C and D) Cell viability of BEAS-2B and RAW 264.7 cells assessed by the CCK-8 assay. ***P < 0.001 versus the control group. ^#^*P* < 0.05 versus the 1,000 μg/ml PS NPs group.

To further validate whether PS NPs-induced apoptosis was mediated by oxidative stress, rescue experiments were performed using N-acetylcysteine (NAC), a classical free radical scavenger [53–55]. BEAS-2B and RAW 264.7 cells were pretreated with NAC (4 mM) for 2 h, followed by exposure to 1,000 μg/ml PS NPs for 24 h. As shown in Fig. 5A and B, NAC pretreatment effectively suppressed the intracellular ROS generation induced by PS NPs in both cell lines. Subsequent CCK-8 assay revealed a prominent restoration of cell viability in NAC-treated cells (Fig. 5C and D). These results demonstrated that oxidative stress played a central role in PS NP-mediated cytotoxicity, and antioxidant supplementation could effectively mitigate nanoparticle-induced damage. This finding aligns with previous reports showing that antioxidant supplement attenuated nanoplastic- or metal-induced toxicity by reestablishing intracellular glutathione (GSH) homeostasis and preserving mitochondrial integrity [56–58]. Importantly, our results suggested that ROS overproduction represents an early and actionable biomarker of nanoplastic exposure, and that redox modulation strategies, such as NAC pretreatment, could serve as potential preventive or therapeutic interventions for nanoplastic-associated pulmonary injury. Together with our hierarchical oxidative stress and inflammation data, these results confirmed that PS NPs-induced pulmonary toxicity followed a redox-dependent cascade, linking environmental nanoplastic exposure to cellular oxidative damage and inflammatory progression.

### PS NPs induced acute and subacute pulmonary injuries in Balb/c mice

**Fig. 6.**
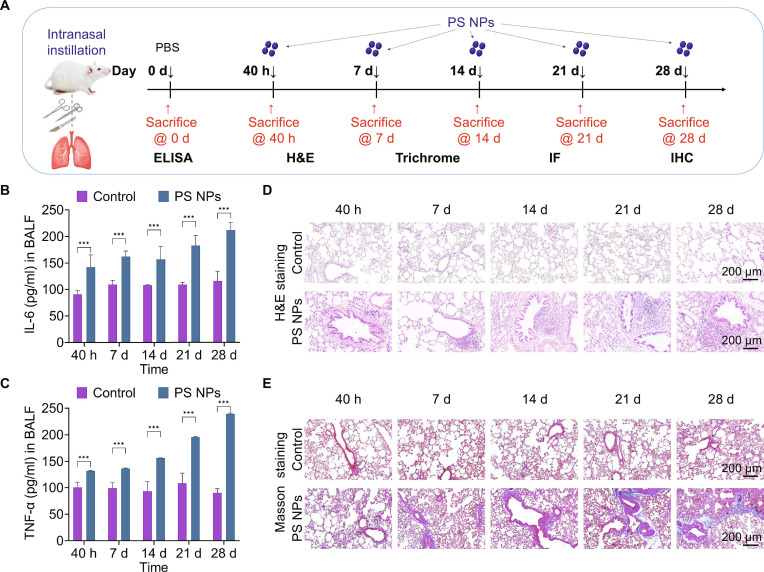
PS NPs induced acute and subacute pulmonary injury in Balb/c mice. (A) Schematics showing the exposure conditions of PS NPs in the murine lung. (B and C) Expression levels of IL-6 and TNF-α in the BALF of Balb/c mice treated with PS NPs. (D and E) Representative images of H&E staining and Masson’s trichrome staining showing the inflammatory cell infiltration and the collagen deposition in the lung tissues. ***P < 0.001 versus the control group.

These in vitro findings suggested that PS NPs may induce acute and subacute pulmonary injury. To validate the in vitro findings and confirm the in vivo pulmonary deposition of PS NPs, we first employed _GF_PS NPs to visualize their distribution in mouse lungs. Frozen lung sections collected at 40 h post-intranasal exposure exhibited distinct green fluorescence signals in the bronchial and alveolar regions (Fig. S22), demonstrating that PS NPs could enter and accumulate in lung tissues during the early stage of exposure. Subsequently, we conducted a 28-d time-gradient exposure study in Balb/c mice via nasal instillation, systematically evaluating PS NPs-induced lung injuries (Fig. 6A). Terminal bronchoalveolar lavage fluid (BALF) and serum cytokine analyses revealed statistically dominant elevations of IL-6 and TNF-α in BALF after sustained PS NP exposure (Fig. 6B and C and Fig. S23). Although IL-6 in serum exhibited an upward trend, TNF-α variations remained statistical insignificance and markedly lower than those data in BALF (Fig. S24), suggesting that the inflammatory responses were predominantly localized in the lung. Elevated levels of these characteristic lung inflammation markers were corroborated by hematoxylin and eosin (H&E) staining, which exhibited notable inflammatory cell infiltration around bronchioles. Prolonged exposure further induced progressive peripheral airway inflammation (Fig. 6D). Masson’s trichrome staining confirmed aggravated fibrotic pathology, consistent with escalating TNF-α levels observed in BEAS-2B cells (Fig. 6E and Fig. S25).

**Fig. 7.**
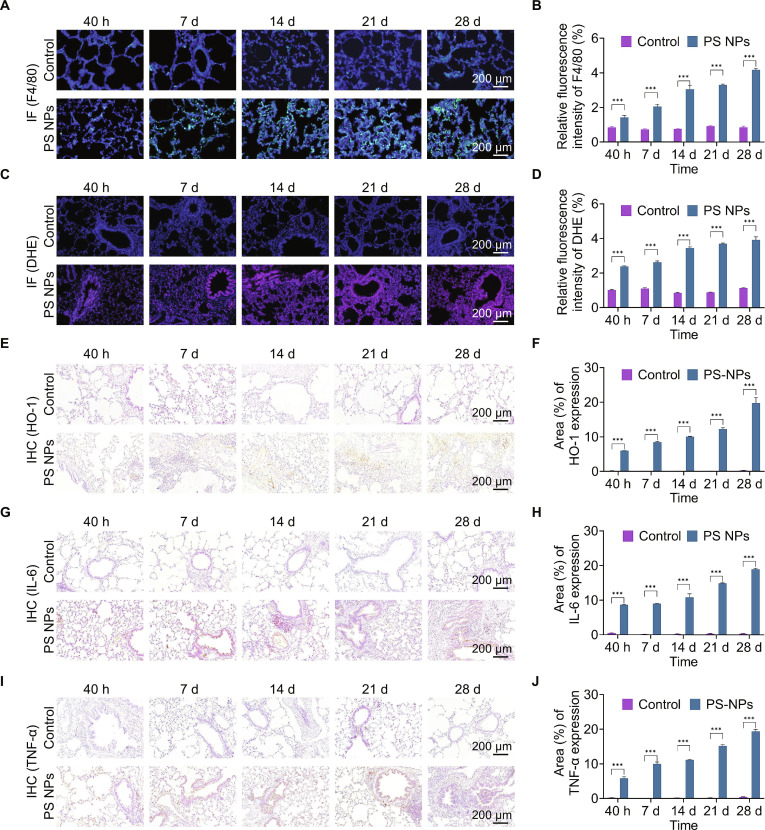
PS NPs induced oxidative stress responses and pulmonary injury in Balb/c mice. (A and B) IF staining and the analysis of relative fluorescence intensity of F4/80 displaying the accumulation of macrophages (green fluorescence) in the lung tissues. (C and D) IF staining and the analysis of relative fluorescence intensity of dihydroethidium (DHE) showing the ROS levels in the lung tissues. (E, G, and I) Representative images of IHC staining showing the expression of HO-1, IL-6, and TNF-α in the lung tissues. (F, H, and J) Analysis of area of HO-1, IL-6, and TNF-α based on IHC staining. ***P < 0.001 versus the control group.

Furthermore, immunofluorescence (IF) staining detected substantial alveolar macrophage recruitment (green fluorescence) to interstitial lung tissue at 40 h post-exposure, with persistent macrophage retention throughout the experimental period (Fig. 7A and B). Aligning with the hierarchical oxidative stress mechanism identified in vitro, sustained ROS elevation in lung tissue from 40 h to 28 d was observed (Fig. 7C and D), supported by immunohistochemical (IHC) data showing substantial HO-1 up-regulation (Fig. 7E and F). Notably, the time-dependent accumulation of IL-6 and TNF-α exhibited strong concordance with in vitro toxicological profiles (Fig. 7G to J). Collectively, our integrated in vitro and in vivo data systematically elucidate a coherent mechanism in which PS NPs-triggered oxidative stress initiates inflammatory cascades, eventually progressing to fibrotic structural lung damage, highlighting the substantial respiratory health risks posed by nanoplastic exposure [59].

### Extended biocompatibility and systemic safety assessment

Beyond pulmonary toxicity, further biosafety evaluations were conducted to assess potential systemic and hematological effects. In vitro hemolysis assays demonstrated that PS NPs induced dose-dependent hemolysis (Fig. S26). Moreover, H&E staining of major organs ( heart, liver, spleen, and kidneys) revealed distinct organ-specific responses (Fig. S27). No structural abnormalities or inflammatory lesions were observed in the heart and spleen, whereas discernible pathological alterations existed in the liver and kidney of mice at higher exposure doses. The liver had mild inflammatory infiltration, primarily localized within the portal areas and central venous regions, without apparent hepatocellular necrosis or structural disruption. Early signs of glomerular injury were noted in the kidney, characterized by mild glomerular atrophy, although tubular architecture remained largely intact. These results indicate that PS NPs-induced toxicity is largely localized to the pulmonary system, with only minor hepatic and renal burden under the tested conditions. Taken together, these findings provide a comprehensive toxicological profile of PS NPs, integrating respiratory, systemic, and hematological endpoints to support a more complete evaluation of their biological safety.

## Conclusion

This study systematically elucidated the mechanisms underlying the respiratory toxicity of PS NPs. In both BEAS-2B and RAW 264.7 cell models, PS NPs induced dose-dependent cytotoxicity through a hierarchical oxidative stress mechanism, manifesting as ROS accumulation, pro-inflammatory cytokine release, and mitochondrial respiratory suppression. Animal experiments further confirmed that prolonged PS NPs exposure caused acute and subacute pulmonary inflammation, time-dependent accumulation of pro-inflammatory cytokines, and fibrotic progression, consistent with in vitro toxicological signatures. This study established a complete evidence chain from dual-cell in vitro models to time-gradient in vivo exposure, enabling full mechanistic delineation from molecular initiating events to terminal pathological outcomes. Our work not only validates the universal applicability of the hierarchical oxidative stress paradigm to emerging contaminants, but also establishes a new paradigm for assessing the respiratory health risks of nanoplastics. More importantly, this study raises a warning that susceptible individuals with long-term plastic exposure should receive regular pulmonary monitoring.

## Materials and Methods

### Materials and reagents

PS NPs (42.1 ± 5.4 nm) and _GF_PS NPs (with the excitation of 488 nm and emission of 518 nm) were purchased from Jiangsu Zhichuan Technology (Changzhou, China). Dipalmitoyl phosphatidylcholine (DPPC) was purchased from Sigma. DMEM, fetal bovine serum (FBS), and PBS were purchased from Procell Life Science & Technology (Wuhan, China). ROS assay kit, mitochondrial superoxide assay kit, mitochondrial membrane potential (JC-1) assay kit, N-acetylcysteine (NAC), and Annexin V–FITC/PI apoptosis detection kit were obtained from Beyotime Biotechnology (Shanghai, China). CCK-8 solutions, Hoechst 33342 staining solution, and fluorescent secondary antibodies were purchased from Boster Biotechnology (Hefei, China). Calcein AM, PI, and enhanced chemiluminescence (ECL) solution were all obtained from KeyGEN BioTECH (Nanjing, China). Primary antibodies (Nrf2, HO-1, F4/80, IL-6, TNF-α, β-actin) were purchased from ABclonal Biotechnology (Wuhan, China). ELISA assay kits were purchased from Meimian Biotechnology, ABclonal Biotechnology, and Feiyue Biotechnology (Wuhan, China). H&E staining kit and Masson’s trichrome staining kit were both provided by Solarbio (Beijing, China).

### Cell culture

BEAS-2B (RRID:CVCL_0168) and RAW 264.7 (RRID:CVCL_0493) cell lines were obtained from the Cell Bank of the Chinese Academy of Sciences (Shanghai, China). The identity of each cell line was authenticated by short tandem repeat (STR) profiling provided by the supplier, showing >90% match with the reference profiles, and neither cell line was listed as misidentified in the International Cell Line Authentication Committee (ICLAC) or Cellosaurus databases. All cell lines were routinely tested and confirmed negative for mycoplasma contamination prior to use. Cells within 3 to 20 passages after thawing were used to minimize genetic drift. Both BEAS-2B and RAW 264.7 cells were cultured in DMEM supplemented with 10% FBS and 1% penicillin–streptomycin (100 U/ml each) in a humidified incubator at 37 °C with 5% CO₂. BEAS-2B and RAW 264.7 cells were selected to represent airway epithelial and immune responses, respectively. This combination of cell models is widely employed in nanoparticle toxicological studies and allows for direct correlation between human-relevant epithelial injury and murine immune responses observed in the in vivo mouse model [35–39]. Cells were passaged every 1 to 2 d using trypsin digestion upon reaching 80% to 90% confluence. 

### Cellular uptake assay

BEAS-2B or RAW 264.7 cells were seeded in 24-well plates overnight. Then, the cells were treated with _GF_PS NPs (1,000 μg/ml) for 24 h. After exposure, the cells were rinsed with PBS and subsequently stained with Hoechst 33342 (5 μM, 10 min) in the dark. Cellular internalization of PS NPs was visualized using a fluorescence microscope (Nikon Ti-S, Japan).

### Cell viability assay

BEAS-2B or RAW 264.7 cells were seeded in 96-well plates overnight. Then, the cells were exposed to PS NPs with different concentrations (0 to 1,000 μg/ml) for 24 h. Then, 10 μl of CCK-8 solution was added to each well and incubated for 2 h. The optical density at 450 nm was measured by SpectraMax iD3.

For live/dead cell staining, BEAS-2B or RAW 264.7 cells were seeded in 24-well plates and cultured overnight. After exposure to PS NPs for 24 h, the cells were stained with calcein AM and PI for 1 h in the dark. Then, the cells were washed with PBS and visualized by a fluorescence microscope (Nikon Ti-S).

For NAC rescue experiments, BEAS-2B and RAW 264.7 cells were pretreated with NAC (4 mM) for 2 h prior to exposure to PS NPs (1,000 μg/ml) for 24 h. After treatment, cell viability was assessed by the same CCK-8 assay protocol described above.

### Cellular ROS detection

BEAS-2B or RAW 264.7 cells maintained in 24-well plates were stimulated by PS NPs with different concentrations (0 to 1,000 μg/ml). After 24 h, cells were incubated with the ROS-sensitive fluorescent probe DCFH-DA (10 μM) for 30 min in complete darkness. Then, the cells were prepared for fluorescence imaging or flow cytometric detection.

For NAC-pretreated groups, cells were incubated with NAC (4 mM) for 2 h prior to PS NPs exposure, and ROS generation was analyzed using the same DCFH-DA staining procedure.

### Mitochondrial superoxide generation and mitochondrial membrane potential assessments

BEAS-2B or RAW 264.7 cells cultured in 24-well plates were treated by PS NPs with different concentrations (0 to 1,000 μg/ml). Following 24-h treatment, cells were incubated with MitoSox Red (5 μM, 20 min) or JC-1 (5 μM, 60 min). Then, cell nuclei were stained with Hoechst 33342 (5 μM, 10 min). Next, the cells rinsed with PBS were visualized by a fluorescence microscope (Nikon Ti-S).

### OCR assessment

The mitochondrial respiration was detected by Seahorse XFe24 analyzers (Agilent Technologies, USA). Briefly, BEAS-2B or RAW 264.7 cells were plated in XFe24 plates overnight. Also, the sensor cartridge was hydrated overnight. Then, cells were cultured with different concentrations of PS NPs (0 to 1,000 μg/ml) for 24 h. Cells were subsequently equilibrated for 1 h at 37 °C in nonbuffered XF basal medium supplemented with 25 mM glucose, 1 mM sodium pyruvate, and 4 mM glutamine prior to the next OCR measurement cycle. Next, the assay proceeded with a series of injections: oligomycin (1.5 μM), carbonyl cyanide 4-trifluoromethoxy phenylhydrazone (1.0 μM), and antimycin A/rotenone (1.0 μM). Finally, the data were normalized against the cell densities with the Seahorse Wave software (Agilent).

### Apoptosis assay

BEAS-2B or RAW 264.7 cells were cultured with PS NPs (0 to 1,000 μg/ml) for 24 h. All procedures were performed strictly according to the manufacturer’s instructions. Prior to analysis, cells were diluted to 1 × 10^6^ cells/ml in 1× binding buffer and analyzed using a flow cytometer (BD FACSCanto II).

### Western blotting analysis

Following 24-h exposure to PS NPs (0 to 1,000 μg/ml), BEAS-2B or RAW 264.7 cells were harvested and lysed. After quantifying total cellular proteins (Bradford assay, 40 μg), they were separated via 10% sodium dodecyl sulfate–polyacrylamide gel electrophoresis (SDS-PAGE) and immunoblotted onto polyvinylidene difluoride (PVDF) membranes. To prevent nonspecific binding, the membranes were saturated in 5% skim milk for 3 h at room temperature. The membranes were then incubated with primary antibodies diluted in blocking buffer at 4 °C overnight, followed by incubation with horseradish peroxidase (HRP)-conjugated secondary antibodies. The membrane was visualized by Tanon 5200 multi-imaging system (Tanon Science & Technology) after washing 3 times with tris-buffered saline with Tween 20 (TBST).

### Enzyme-linked immunosorbent assay

BEAS-2B or RAW 264.7 cells were incubated with different concentrations of PS NPs for 24 h. Culture supernatants were collected by centrifugation (2,500 rpm, 15 min, 4 °C) to test the pro-inflammatory cytokines (IL-6, IL-8, and TNF-α) levels using commercial ELISA kits. The colorimetric intensity at 450 and 570 nm was measured using a microplate reader (SpectraMax iD3).

### Animal experiment

Male Balb/c mice (6 weeks old) were purchased from Sibef (Beijing) Biotechnology. All animals were obtained from the same breeding batch and were 4 weeks old upon arrival. After a 2-week acclimation period under standardized housing conditions, the animal experiments were initiated when the mice reached 6 weeks of age. All animals were housed in a specific pathogen-free (SPF) facility under a 12/12-h light/dark cycle with free access to standard laboratory chow and autoclaved water. All animal procedures were conducted under a protocol approved by the Animal Research Ethics Committee of Shanxi Medical University (ethical approval no.: SYDL2025012). Mice were randomly divided into the 6 groups (*n* = 6 per group): 0 d, 40 h, 7 d, 14 d, 21 d, and 28 d. PS NPs were administered via intranasal instillation at a dose of 5 mg/kg, which was selected according to previously reported studies on PS NPs-induced pulmonary toxicity in rodents [25,60–63]. Briefly, after mild anesthesia with inhaled isoflurane, each mouse received 20 μl of PS NPs suspension. As a negative control, 20 μl of PBS containing 0.6 mg/ml mouse serum albumin and 10 μg/ml DPPC (pH 7.4) was used to treat the mice. At designated time points, mice were euthanized, and major organs (lungs, heart, liver, spleen, kidneys) were collected for further analyses.

### Fluorescence tracking of PS NPs in vivo

To confirm the pulmonary localization of PS NPs, _GF_PS NPs were administrated to additional groups of Balb/c mice under the same intranasal instillation protocol (5 mg/kg). At 40 h post-exposure, lungs were harvested and immediately embedded in optimal counting temperature compound (OCT) for cryosectioning. Frozen sections (20 μm) were prepared to preserve fluorescence and examined using a fluorescence microscope (Nikon Ti-S, Japan).

### BALF collection and analysis

After left bronchus ligation, the right lung was lavaged (3×) with PBS (27 ml/kg) via tracheal cannulation. BALF supernatant was collected post-centrifugation and stored at −80 °C for multiplex ELISA analysis.

### Blood analysis

Standard blood collection procedures were followed to obtain serum and plasma from Balb/c mice. Samples were stored at −80 °C. Commercial ELISA kits were used to measure IL-6 and tumor necrosis.

### Hemocompatibility test

Fresh whole blood was collected from healthy Balb/c mice using heparinized tubes. Red blood cells (RBCs) were isolated by centrifugation (3,000 rpm, 10 min) and washed 3 times with PBS. Subsequently, RBC suspensions (2%) were incubated with PS NPs at concentrations of 0 to 1,000 μg/ml at 37 °C for 3 h. Deionized water and PBS were used as the positive control and negative control, respectively. After centrifugation, the absorbance of the supernatant at 541 nm was measured to calculate the hemolysis ratio.

The *hemolysis percentage* was calculated according to the following formula:

$$\text{Hemolysis}\;(\%)=\frac{A_{\text{sample}}-A_{\text{PBS}}}{A_{{\text{ddH}}_{2}O}-A_{\text{PBS}}}\times 100\%$$ (1)

### Histopathological analysis

The left lungs, heart, liver, kidneys, and spleen were collected, sectioned into small pieces (4 μm), and fixed in 4% paraformaldehyde. Following dehydration in graded ethanol and clearing in xylene, tissues were embedded in paraffin. H&E staining, Masson’s trichrome staining, IF staining, and IHC analysis were performed to image using optical microscopy (Nikon Ti-S).

### Statistical analysis

All statistical parameters are presented as the mean ± standard deviation (SD). Statistical significance among multiple groups was assessed using one-way analysis of variance (ANOVA), while comparisons between 2 groups were conducted using Student’s *t* test by GraphPad Prism software (version 10.1.2). *P* value of less than 0.05 was considered statistically significant versus control group.

## Data Availability

The datasets generated and/or analyzed during the current study are available within the article and its Supplementary Materials, which includes raw data for figures and statistical analyses. Due to confidentiality, any data not presented here can be made available subject to a nondisclosure agreement.
