# Supplementary material for: Unveiling the Pulmonary Toxicity of Polystyrene Nanoplastics: A Hierarchical Oxidative Stress Mechanism Driving Acute–Subacute Lung Injury
Source: Research (Wash D C). 2025 Nov 24;8:0995. doi: 10.34133/research.0995 (PMC12641161; doi:10.34133/research.0995)
Supplement: Supplementary 1 — Figs. S1 to S27 [file research.0995.f1.docx]

**Supplementary Materials**

Unveiling the Pulmonary Toxicity of Polystyrene Nanoplastics: A Hierarchical Oxidative Stress Mechanism Driving Acute-Subacute Lung Injury

Xianyi Tang^1#^, Lu Lu^2#^, Yuqing Sun^1^, Aiyun Li^1^, Wanzhen Su^1^, Jimin Cao^1^*, Xiao Zhang^3^*, Xi Liu^4^*, Yanlin Feng^1^*

^1^ Department of Cardiology, the First hospital of Shanxi Medical University, and Key Laboratory of Cellular Physiology at Shanxi Medical University, Ministry of Education, Taiyuan, 030001, China.

^2^ Institute of NBC Defence, PLA Army, Beijing, 102205, China.

^3^ Second Clinical Medical College, School of Pharmacy and Key Laboratory of Cellular Physiology, Shanxi Medical University, Taiyuan, 030001, China.

^4^ Medical Innovation Research Division, Chinese PLA General Hospital, Beijing, 100048, China.

^#^ These authors contributed equally to this work.

^*^ Correspondence to:

Jimin Cao, Department of Cardiology, the First hospital of Shanxi Medical University, and Key Laboratory of Cellular Physiology at Shanxi Medical University, Ministry of Education, Taiyuan, 030001, China, caojimin@sxmu.edu.cn.

Xiao Zhang, Second Clinical Medical College, School of Pharmacy and Key Laboratory of Cellular Physiology, Shanxi Medical University, Taiyuan, Shanxi 030001, China, zhangxiao24@a.sxmu.edu.cn.

Xi Liu, Medical Innovation Research Division, Chinese PLA General Hospital, Beijing, 100048, China, liuxipla@163.com.

Yanlin Feng, Department of Cardiology, the First hospital of Shanxi Medical University, and Key Laboratory of Cellular Physiology at Shanxi Medical University, Ministry of Education, Taiyuan, 030001, China, feng@sxmu.edu.cn.


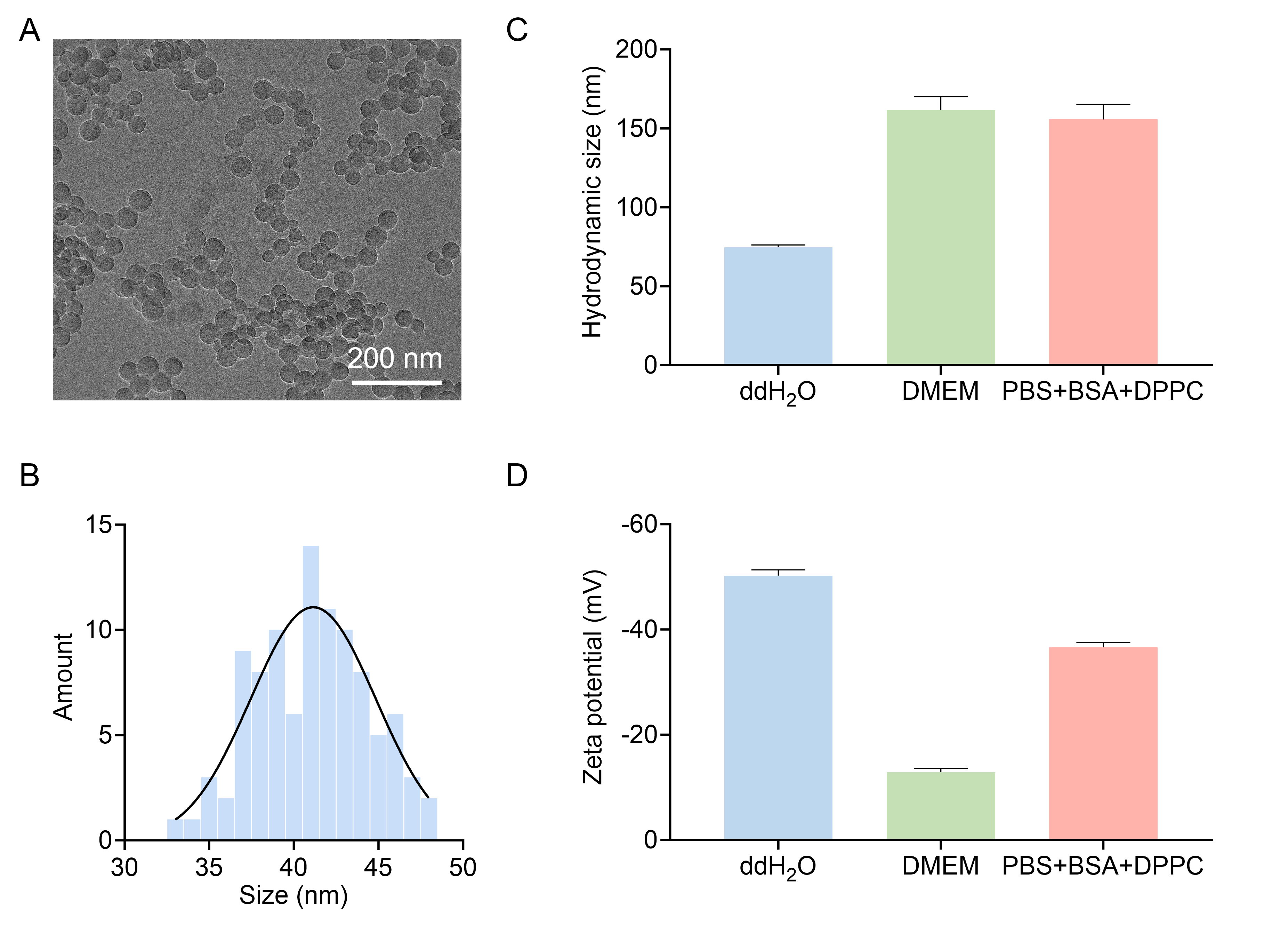


**Figure S1.** (A) Representative TEM images of _GF_PS NPs. (B) Size distribution of _GF_PS NPs. (C and D) Hydrodynamic sizes and Zeta potential of _GF_PS NPs in distilled water, DMEM, and surrogate lung lining fluid.


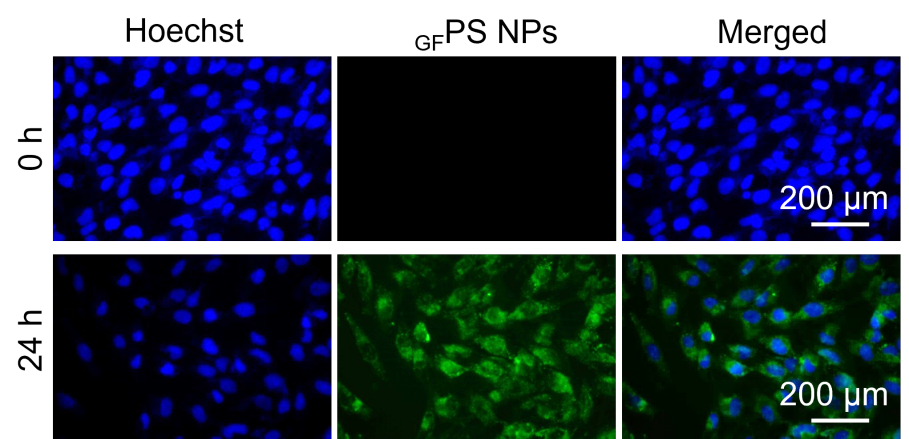


**Figure S2.** Cellular uptake of _GF_PS NPs in BEAS-2B cells after 24 h co-incubation.


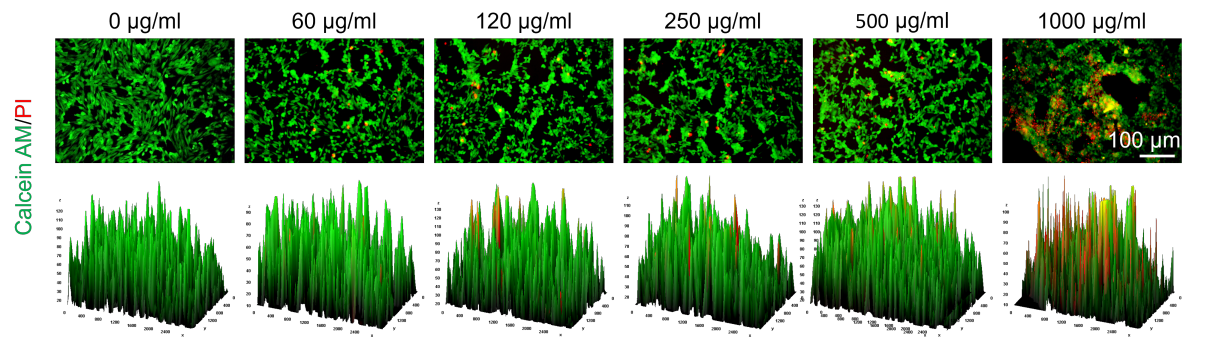


**Figure S3.** Live/dead staining and corresponding 3D surface plots indicating the cell viability of BEAS-2B cells treated with PS NPs.


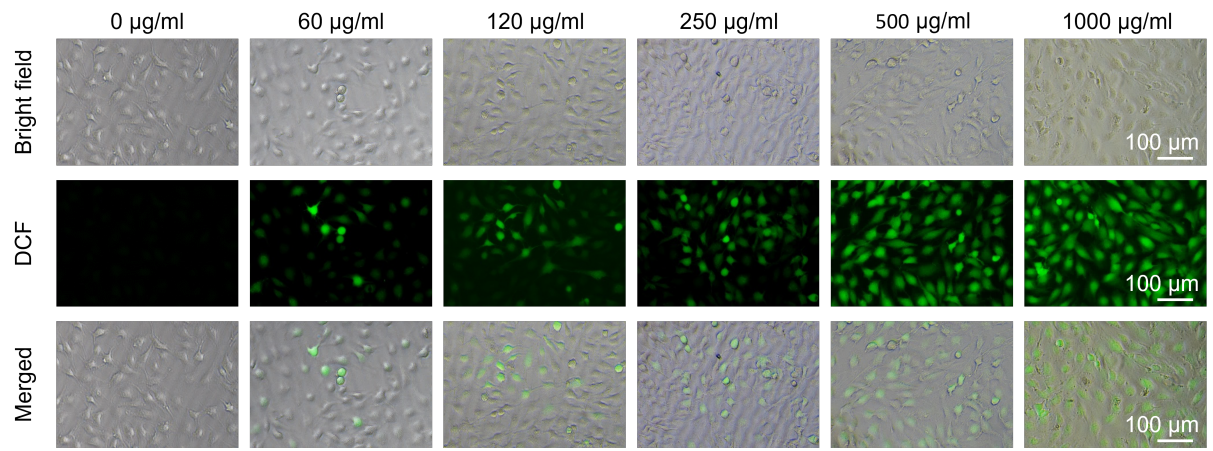


**Figure S4.** Cellular ROS level in PS NPs-treated BEAS-2B cells based on DCF test.


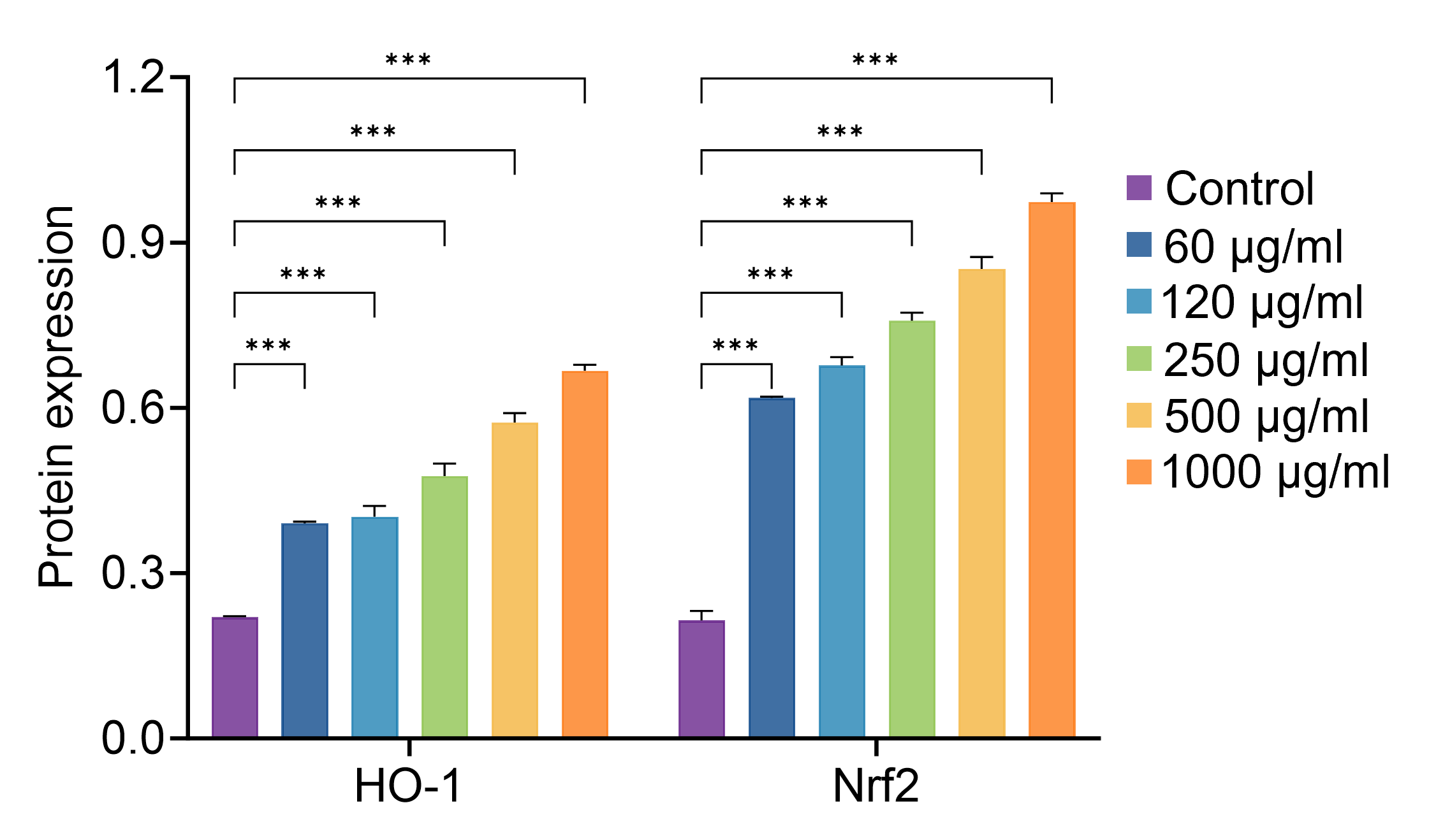


**Figure S5.** Quantitative analysis of protein expression of HO-1 and Nrf2 in BEAS-2B cells treated with PS-NPs. ****P* < 0.001 compared to the 0 μg/ml group.


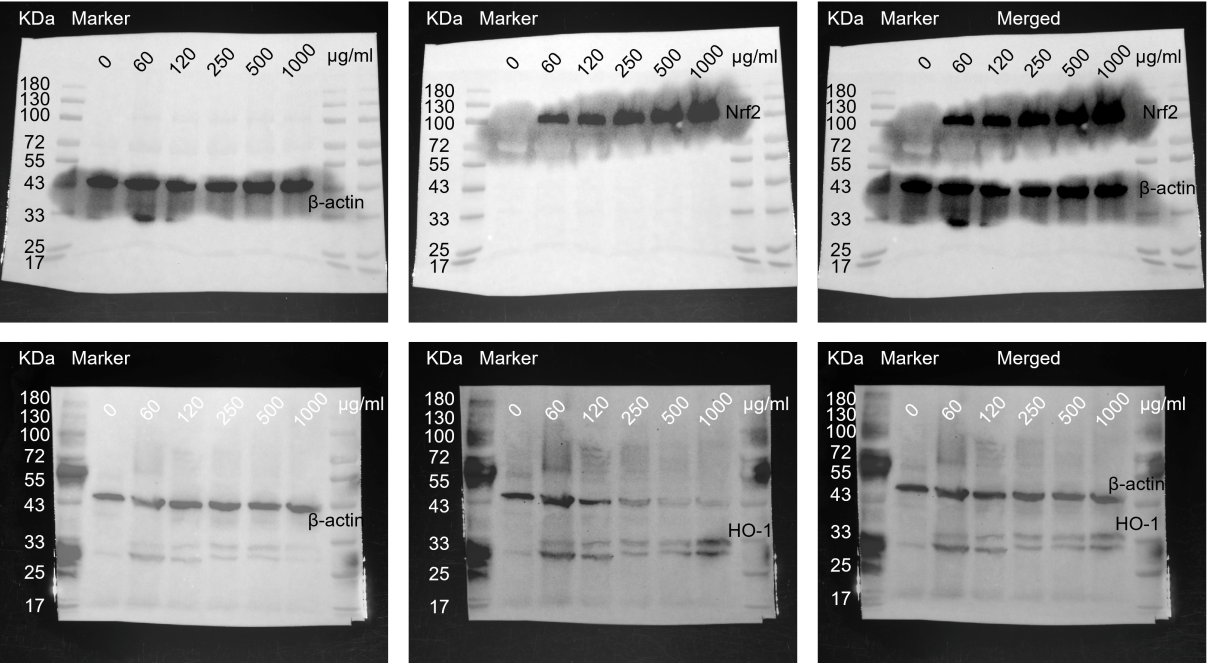


**Figure S6.** The raw data of protein expression of HO-1 and Nrf2 in BEAS-2B cells treated with PS-NPs.


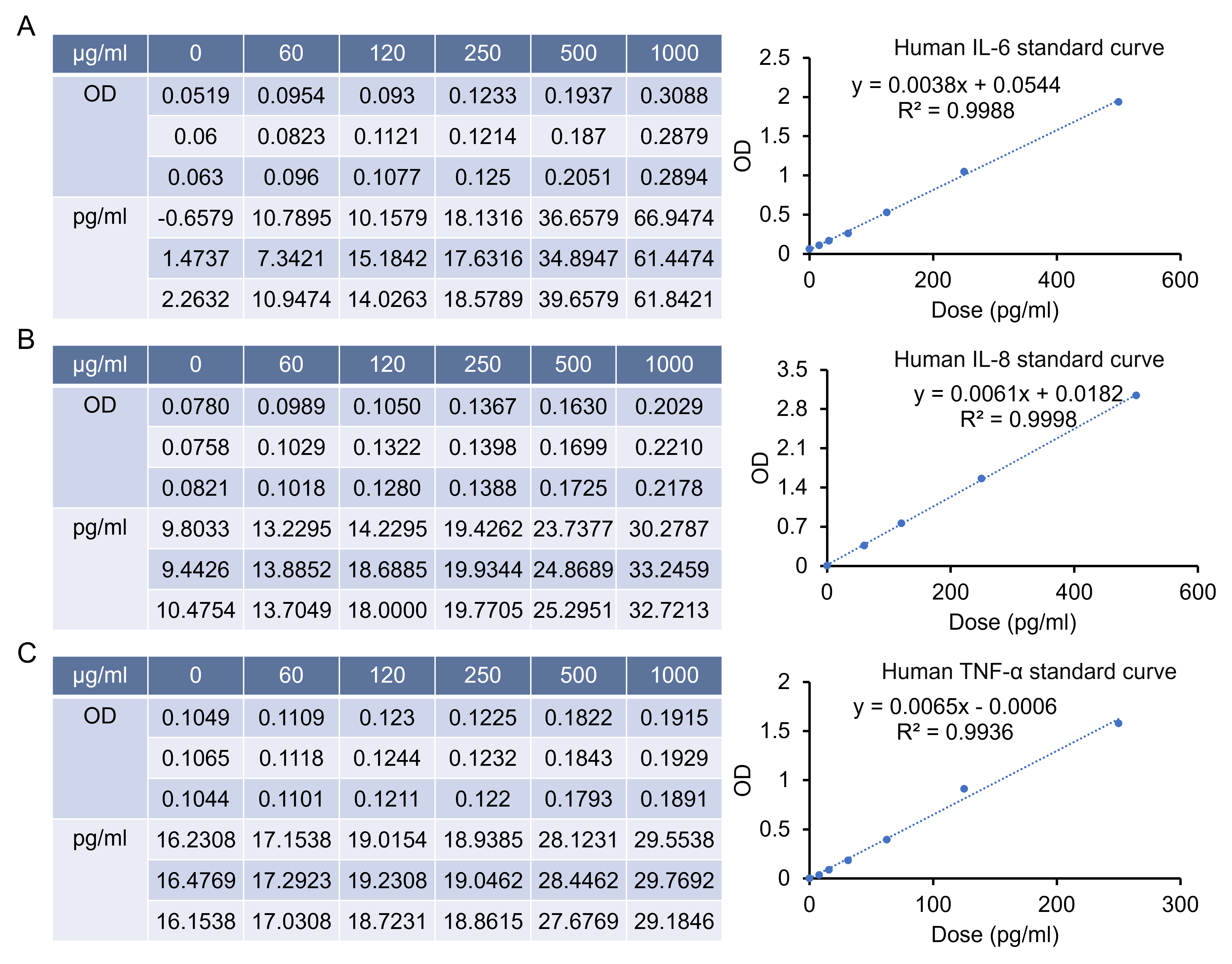


**Figure S7.** ELISA assay assessing the expression of IL-6 (A), IL-8 (B) and TNF-α (C) in BEAS-2B cells treated with PS NPs.


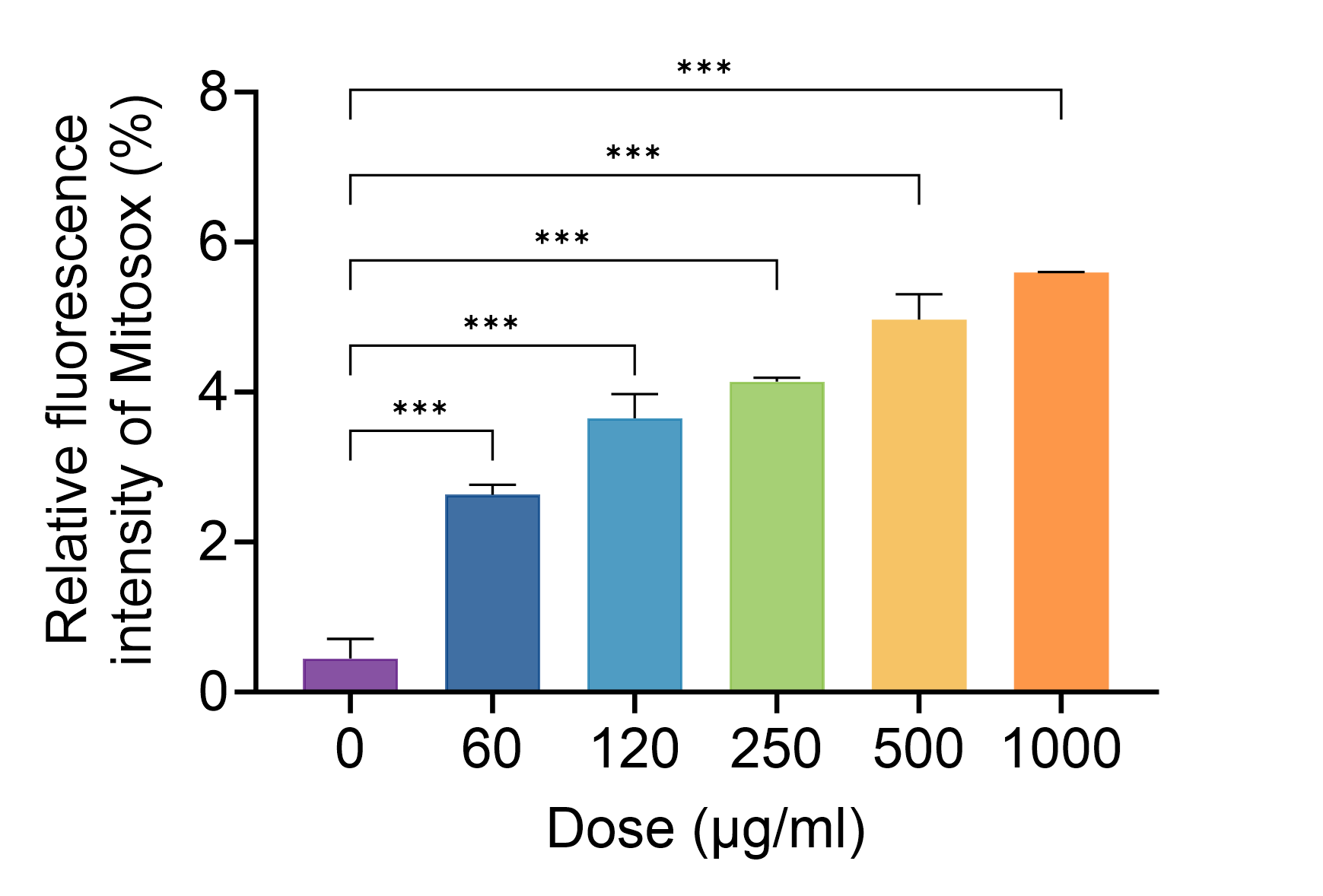


**Figure S8.** Semi-quantitative analysis of MitoSox Red in BEAS-2B cells treated with PS NPs. ****P* < 0.001 compared to the 0 μg/ml group.


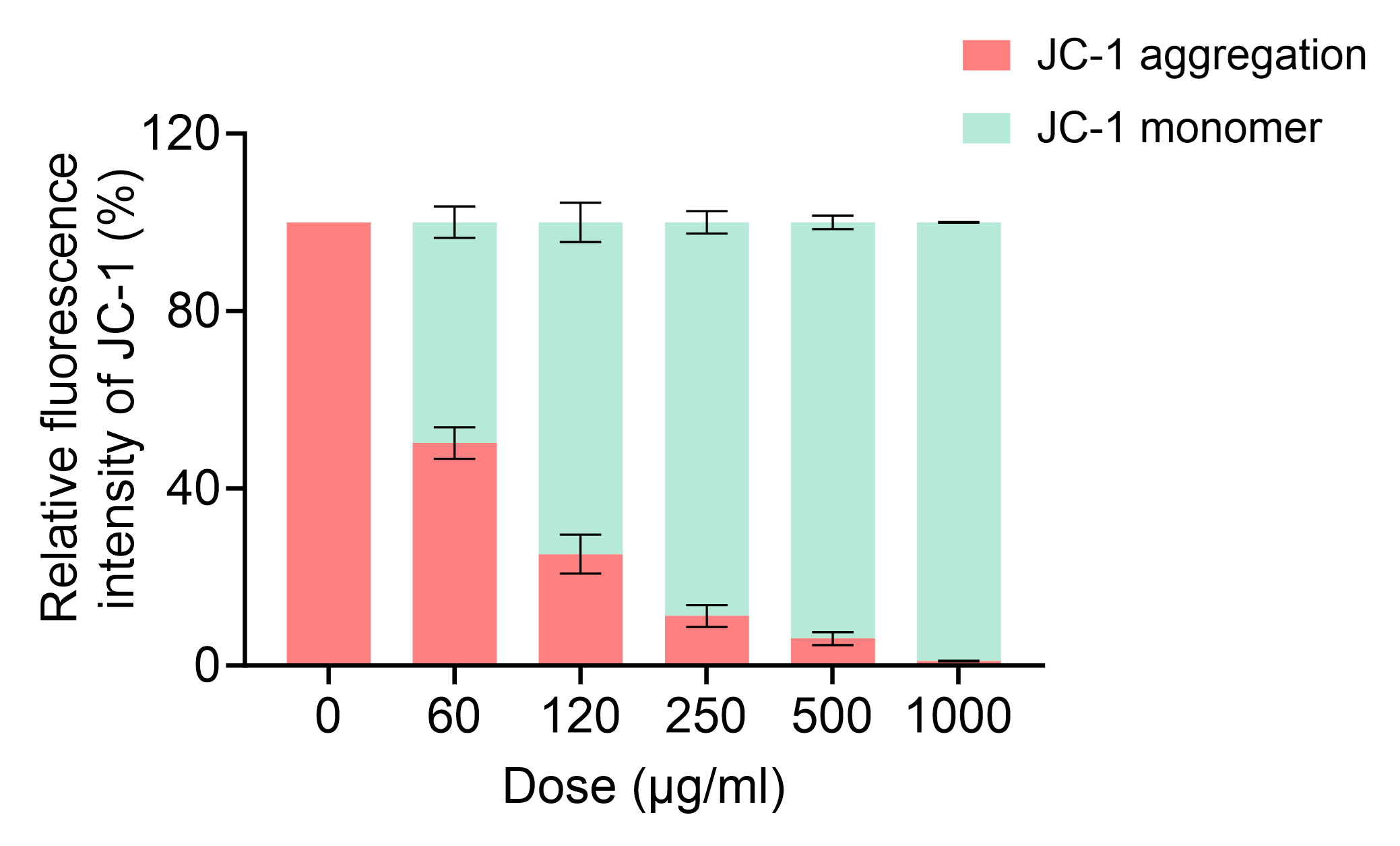


**Figure S9.** Semi-quantitative analysis of JC-1 monomer and JC-1 aggregation in BEAS-2B cells treated with PS NPs.


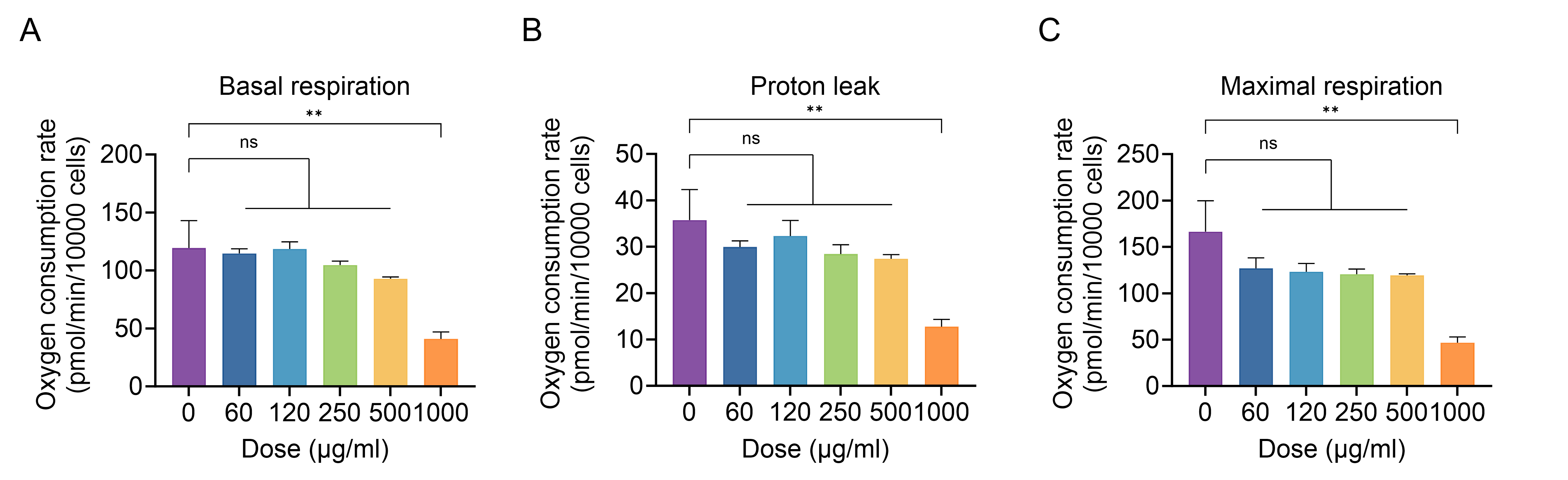


**Figure S10.** Relative changes in key parameters of mitochondrial function in BEAS-2B cells, including (A) basal respiration, (B) proton leak, and (C) maximal respiration in BEAS-2B cells treated with PS NPs. ns is not significant, ***P* < 0.01 compared to the 0 μg/ml group.


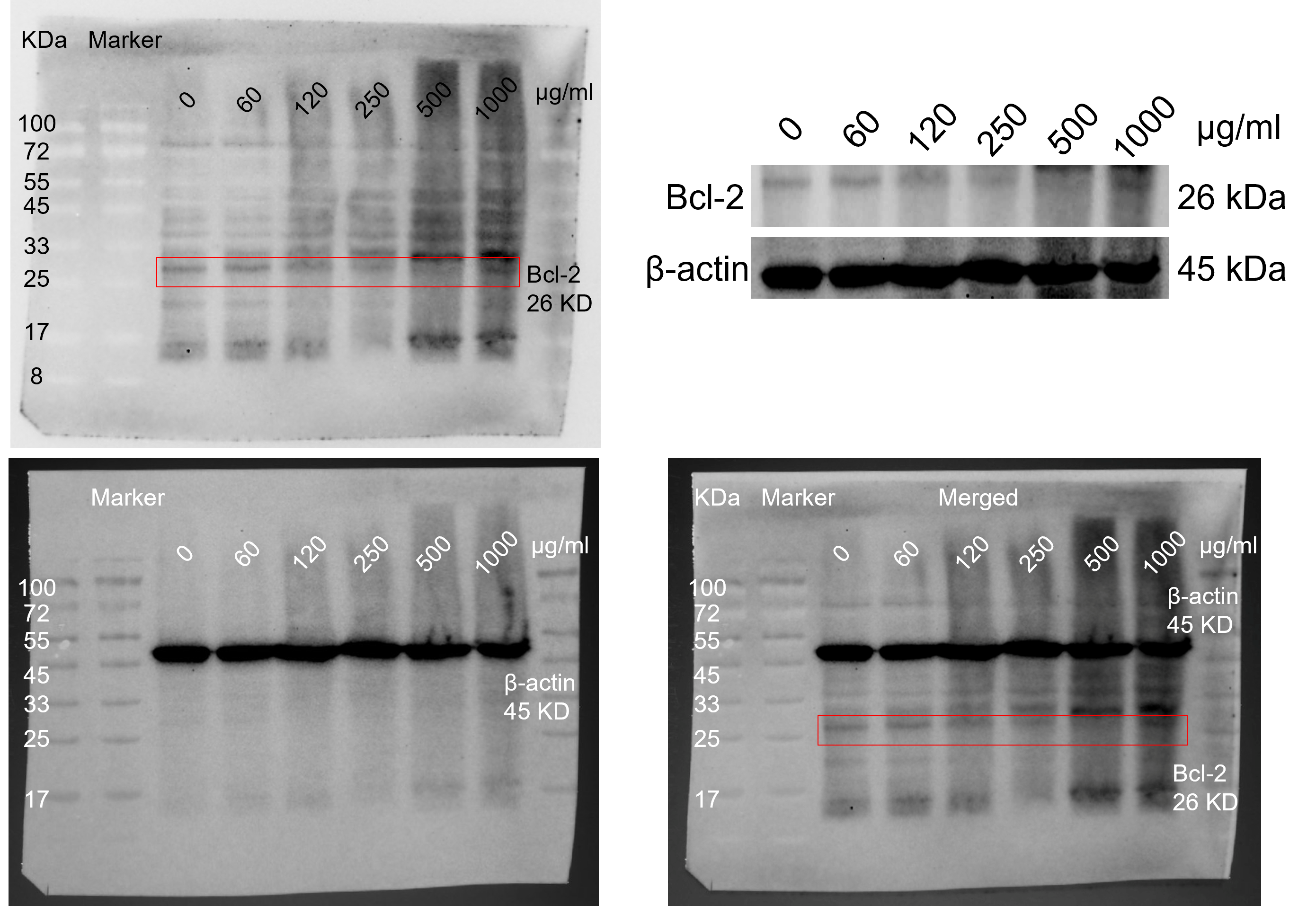


**Figure S11.** The raw data of protein expression of Bcl-2 in BEAS-2B cells treated with PS-NPs.


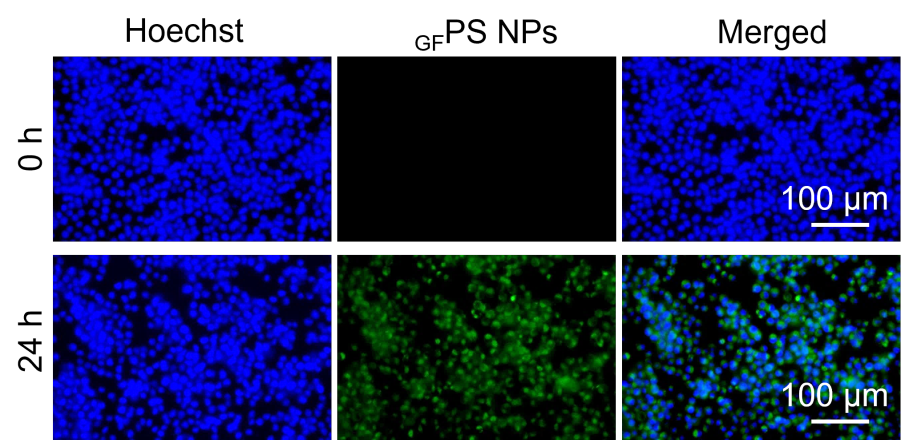


**Figure S12.** Cellular uptake of _GF_PS NPs in RAW 264.7 cells after 24 h co-incubation.


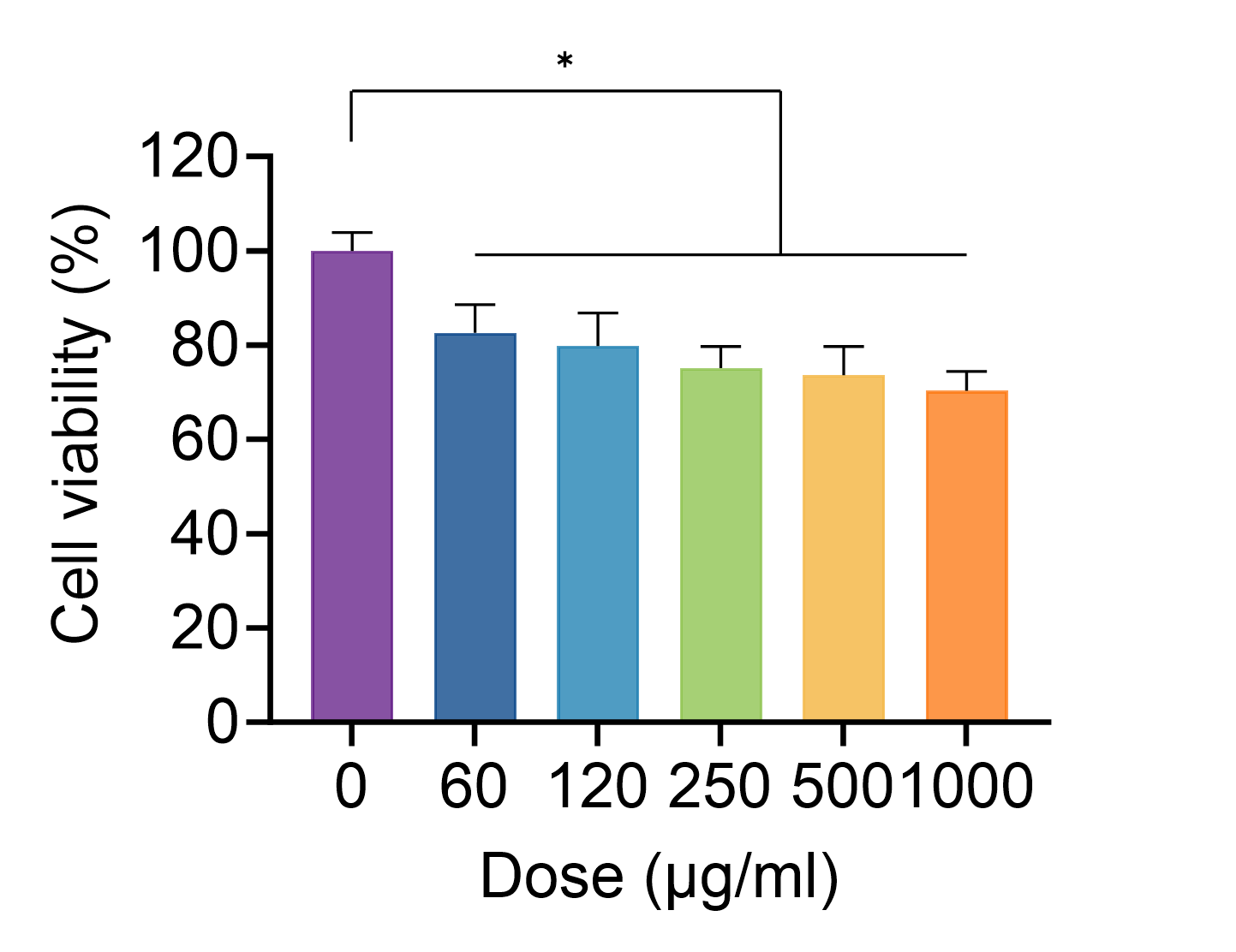


**Figure S13.** CCK-8 assay indicating the cytotoxicity of PS NPs in RAW 264.7 cells treated with PS NPs. **P* < 0.05 compared to the 0 μg/ml group.


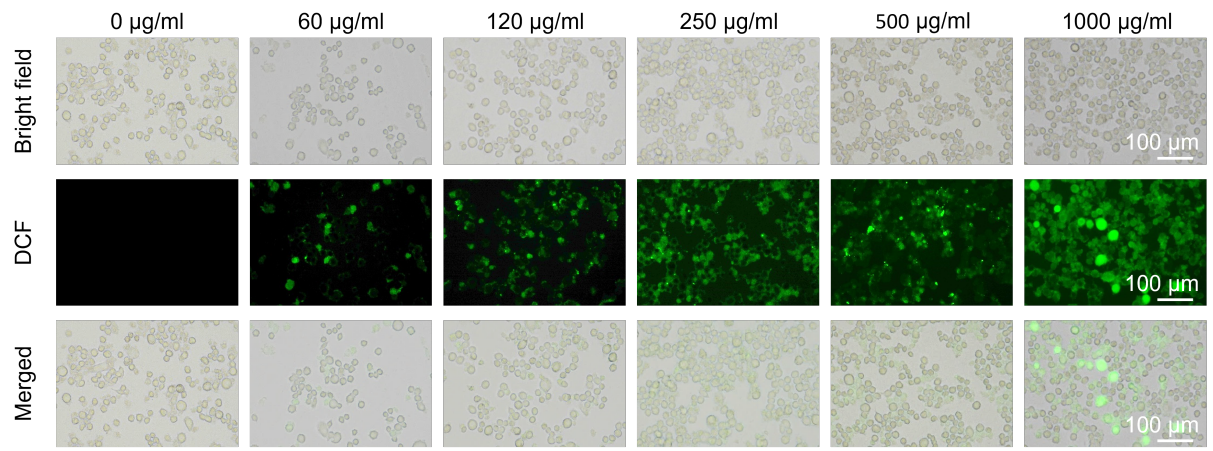


**Figure S14.** Cellular ROS level based on DCF test NPs in RAW 264.7 cells treated with PS NPs.


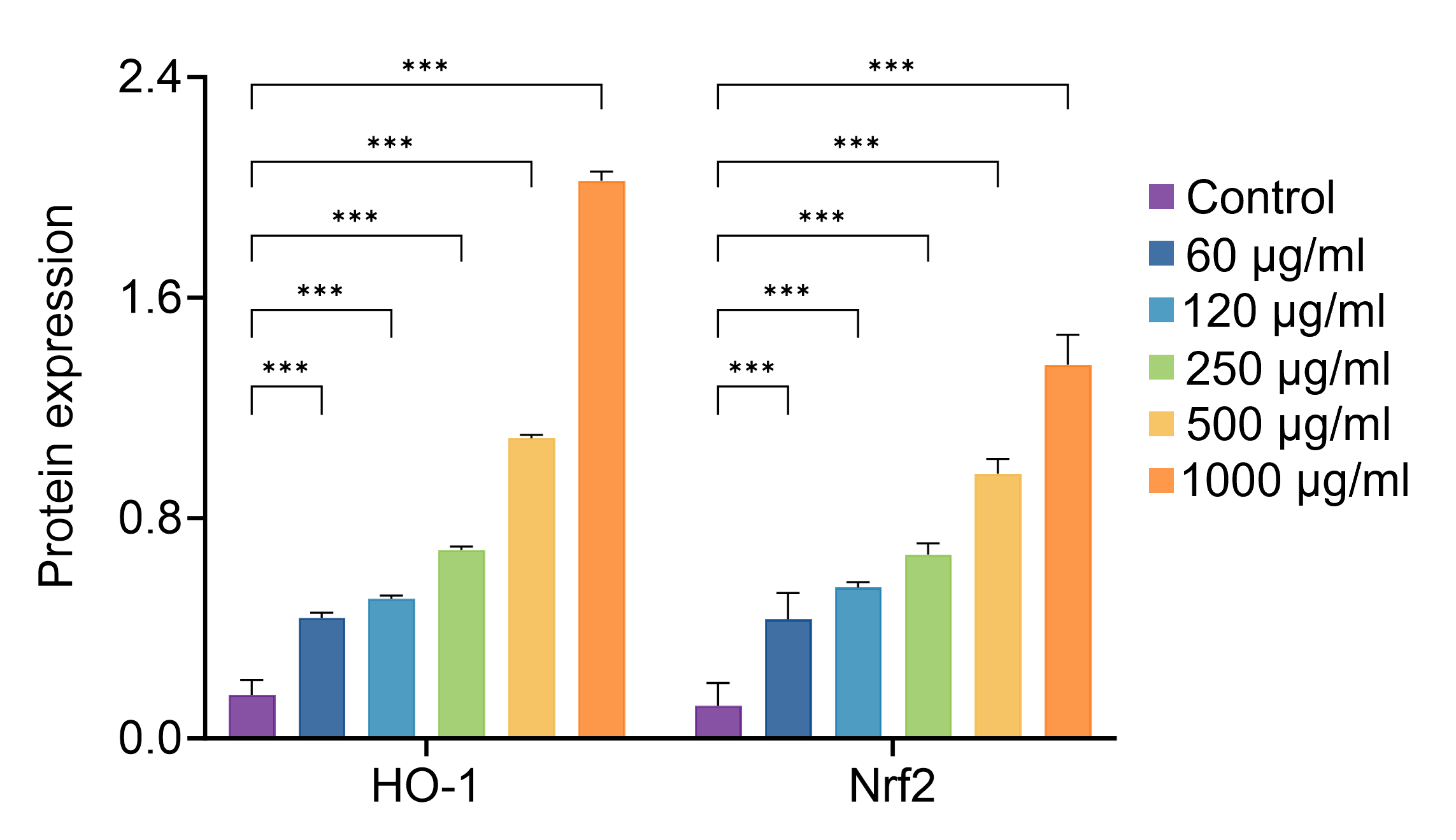


**Figure S15.** Quantitative analysis of protein expression of HO-1 and Nrf2 in Raw 264.7 cells treated with PS-NPs. ****P* < 0.001 compared to the 0 μg/ml group.


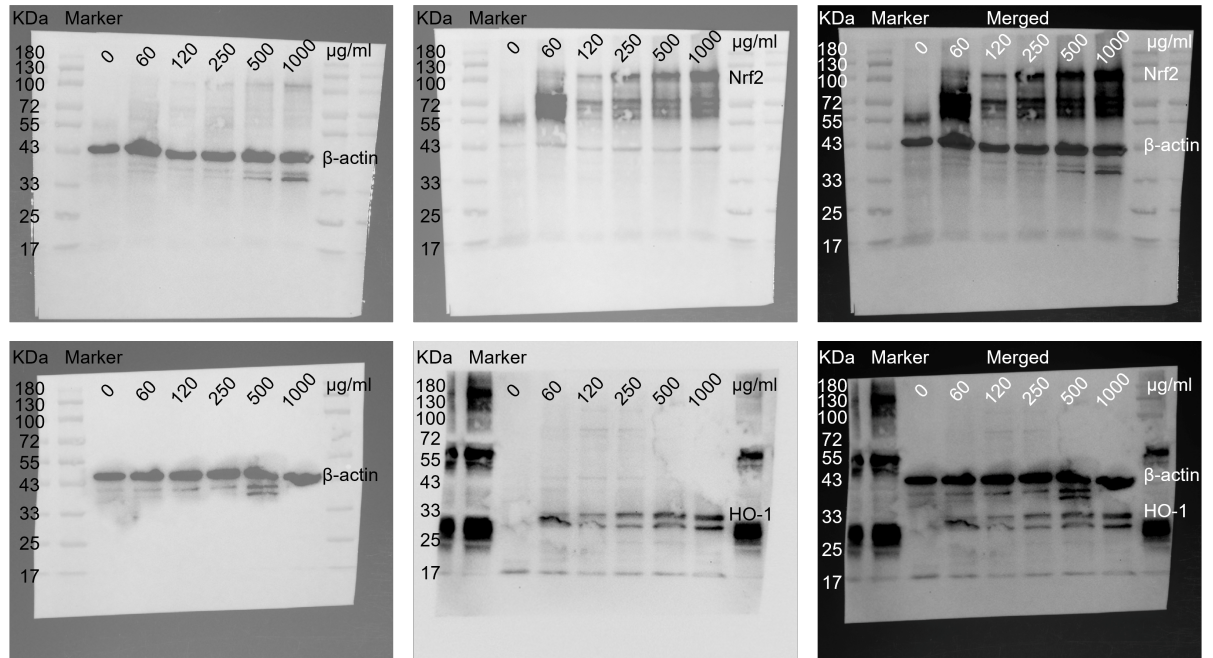


**Figure S16.** The raw data of protein expression of HO-1 and Nrf2 in Raw 264.7 cells treated with PS-NPs.


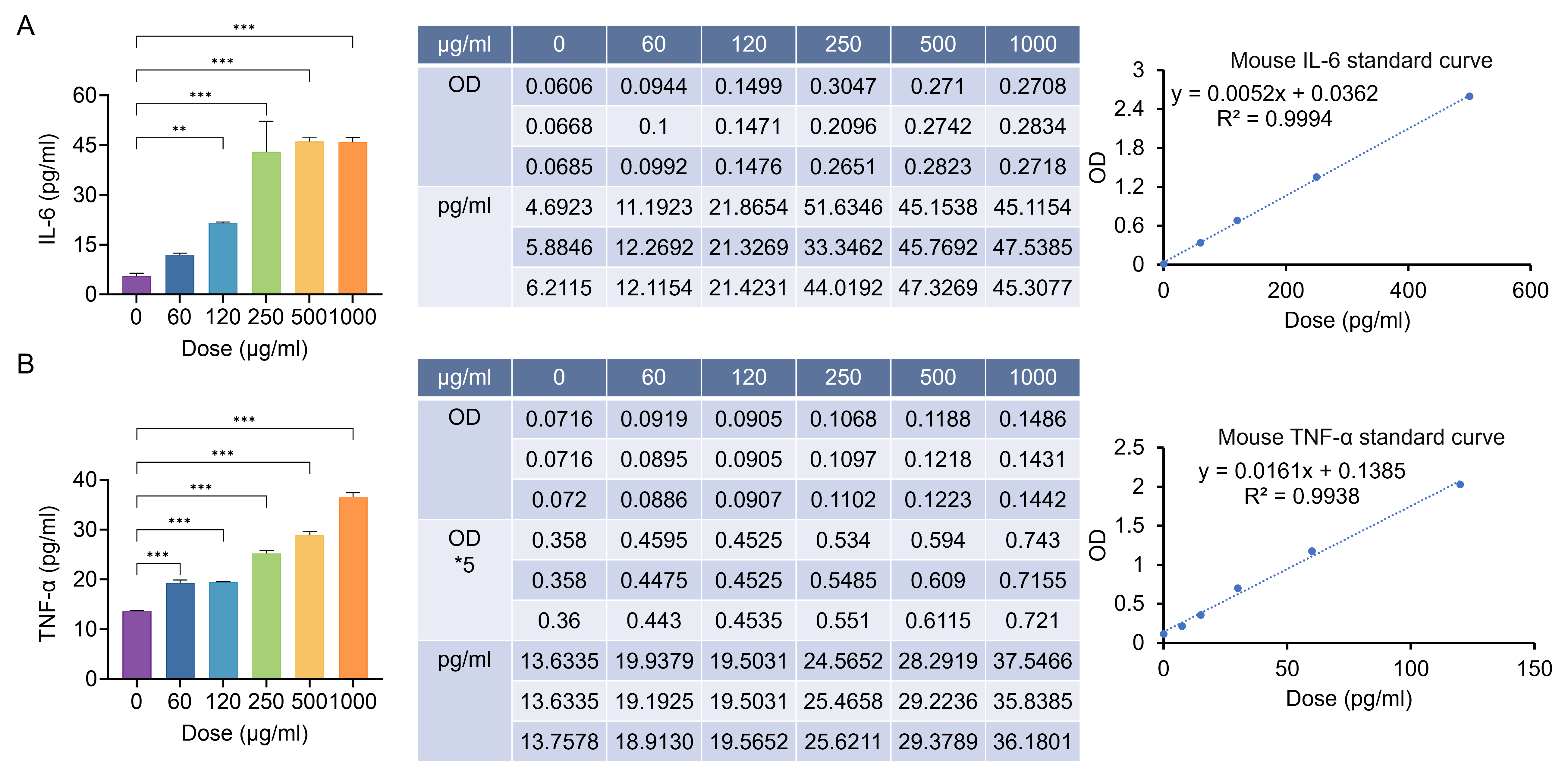


**Figure S17.** ELISA assay assessing the expression of IL-6 (A) and TNF-α (B) in RAW 264.7 cells treated with PS NPs. ***P* < 0.01, ****P* < 0.001 compared to the 0 μg/ml group.

**
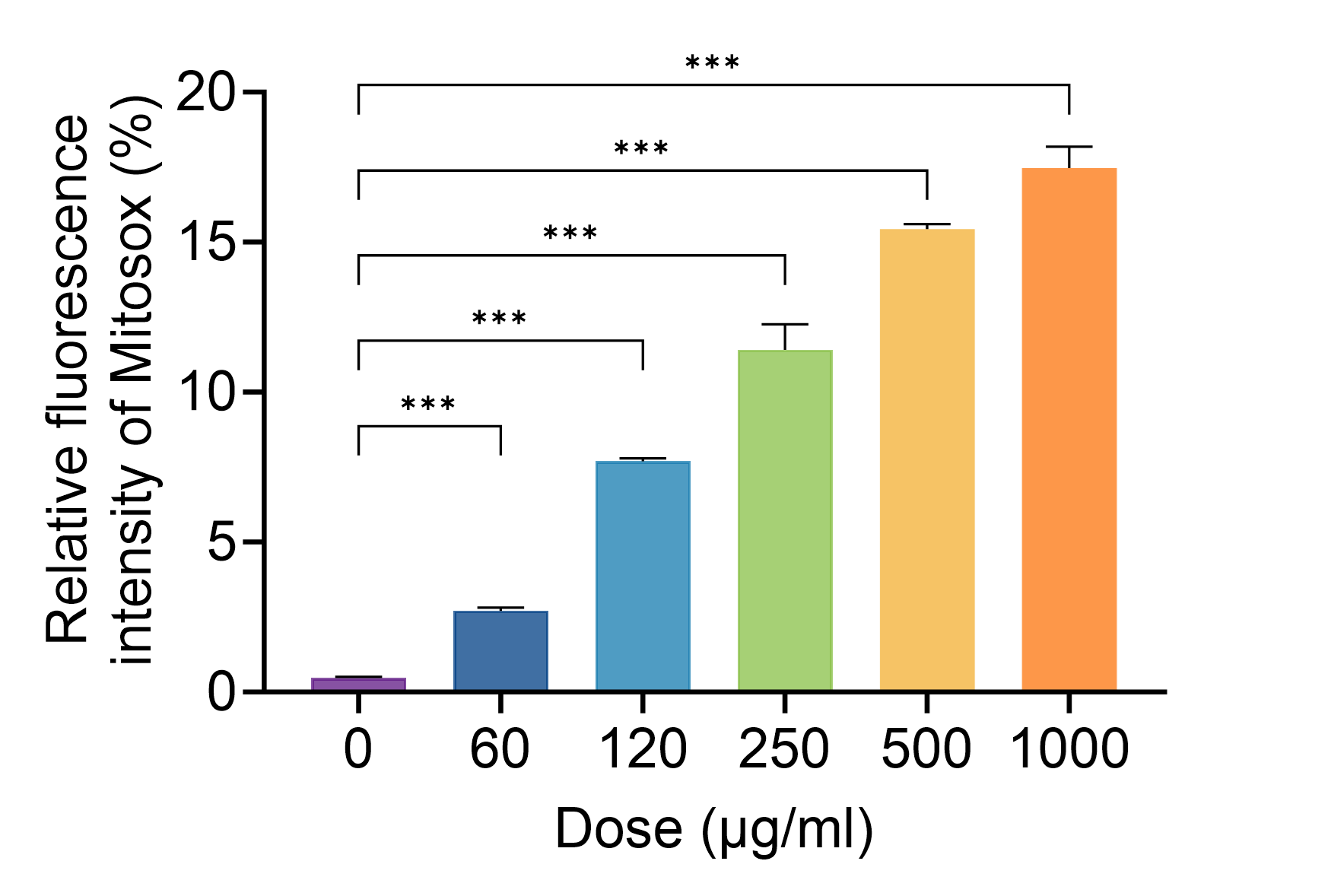
**

**Figure S18.** Semi-quantitative analysis of MitoSox Red in RAW 264.7 cells treated with PS NPs. ****P* < 0.001 compared to the 0 μg/ml group.


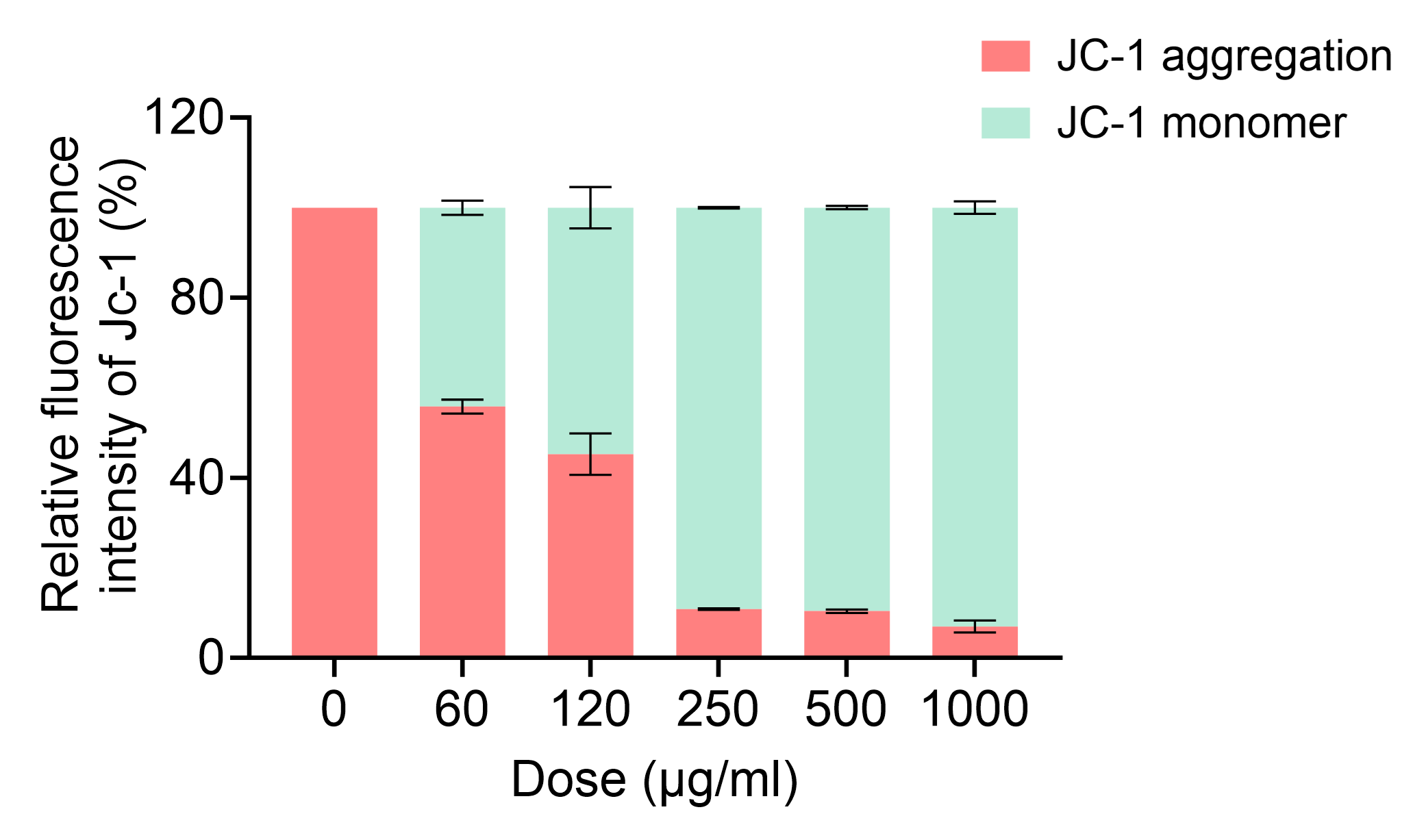


**Figure S19.** Corresponding semi-quantitative analysis of JC-1 monomer and JC-1 aggregation in RAW 264.7 cells treated with PS NPs.


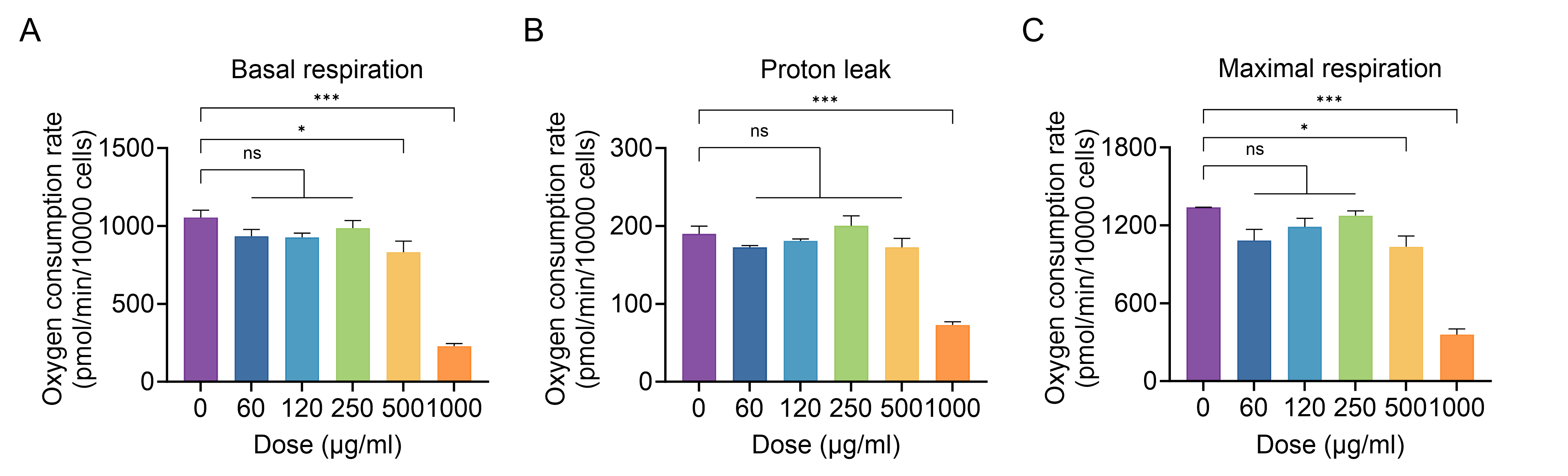


**Figure S20.** Relative changes in key parameters of mitochondrial function in RAW 264.7 cells, including (A) basal respiration, (B) proton leak and (C) maximal respiration in RAW 264.7 cells treated with PS NPs. ns is not significant, **P*< 0.05, ****P*< 0.001 compared to the 0 μg/ml group.


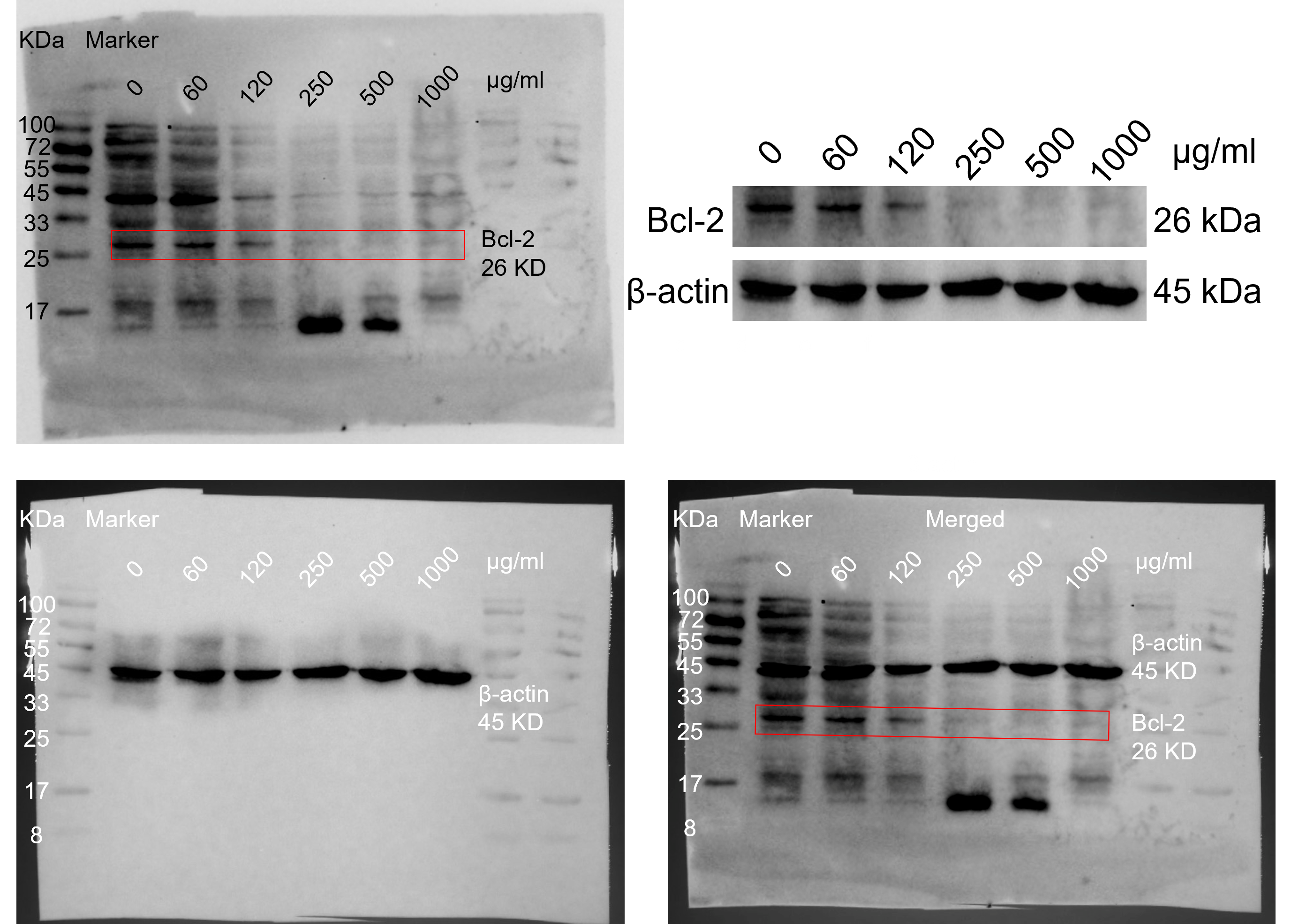


**Figure S21.** The raw data of protein expression of Bcl-2 in RAW 264.7 cells treated with PS-NPs.


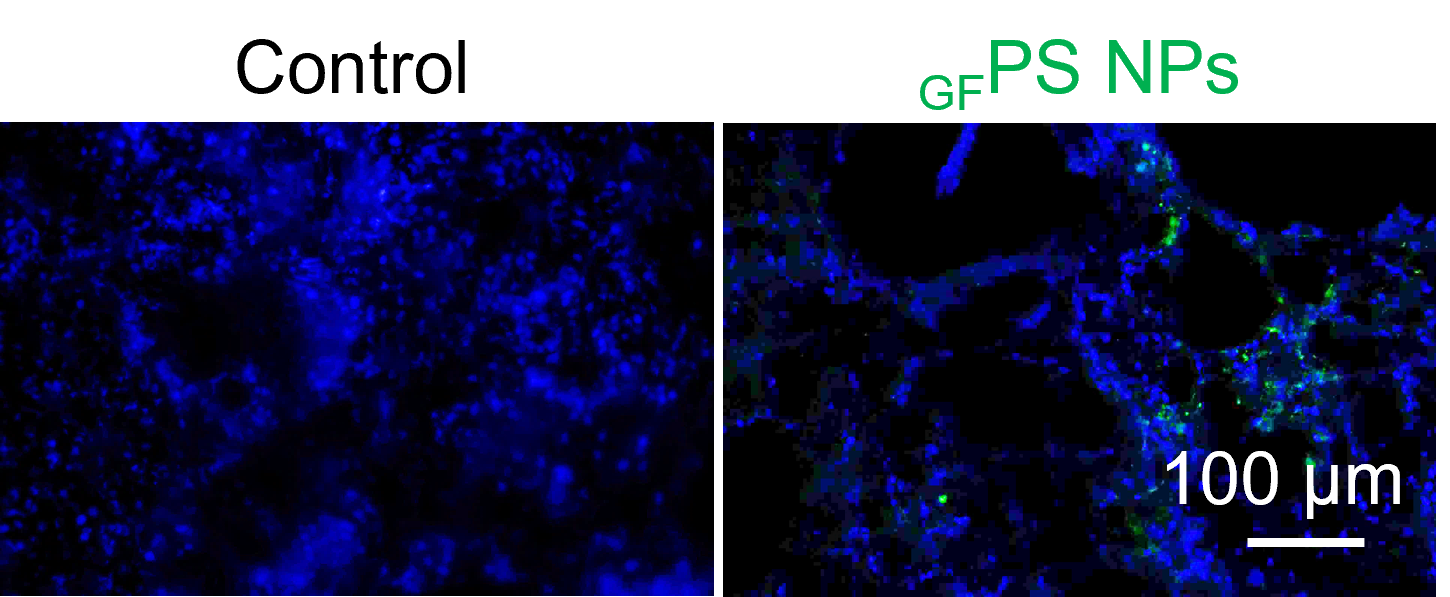


**Figure S22.** Representative fluorescence images of frozen lung sections collected 40 h after intranasal exposure to _GF_PS NPs (5 mg/kg). Cell nuclei were stained with Hoechst 33342 (blue).


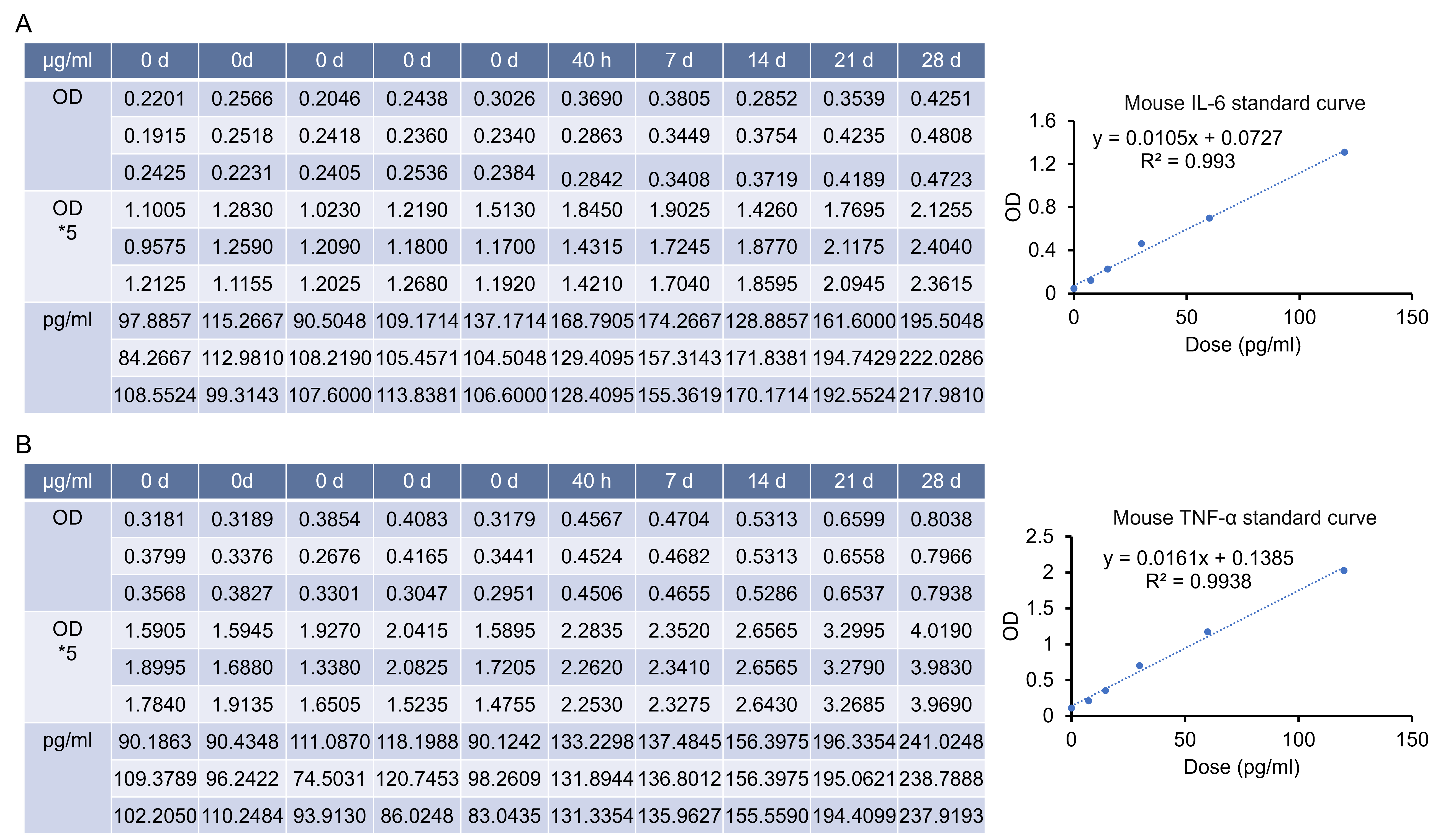


**Figure S23.** ELISA assay assessing the expression levels of IL-6 (A) and TNF-α (B) in the BALF of Balb/c mice treated with PS NPs.


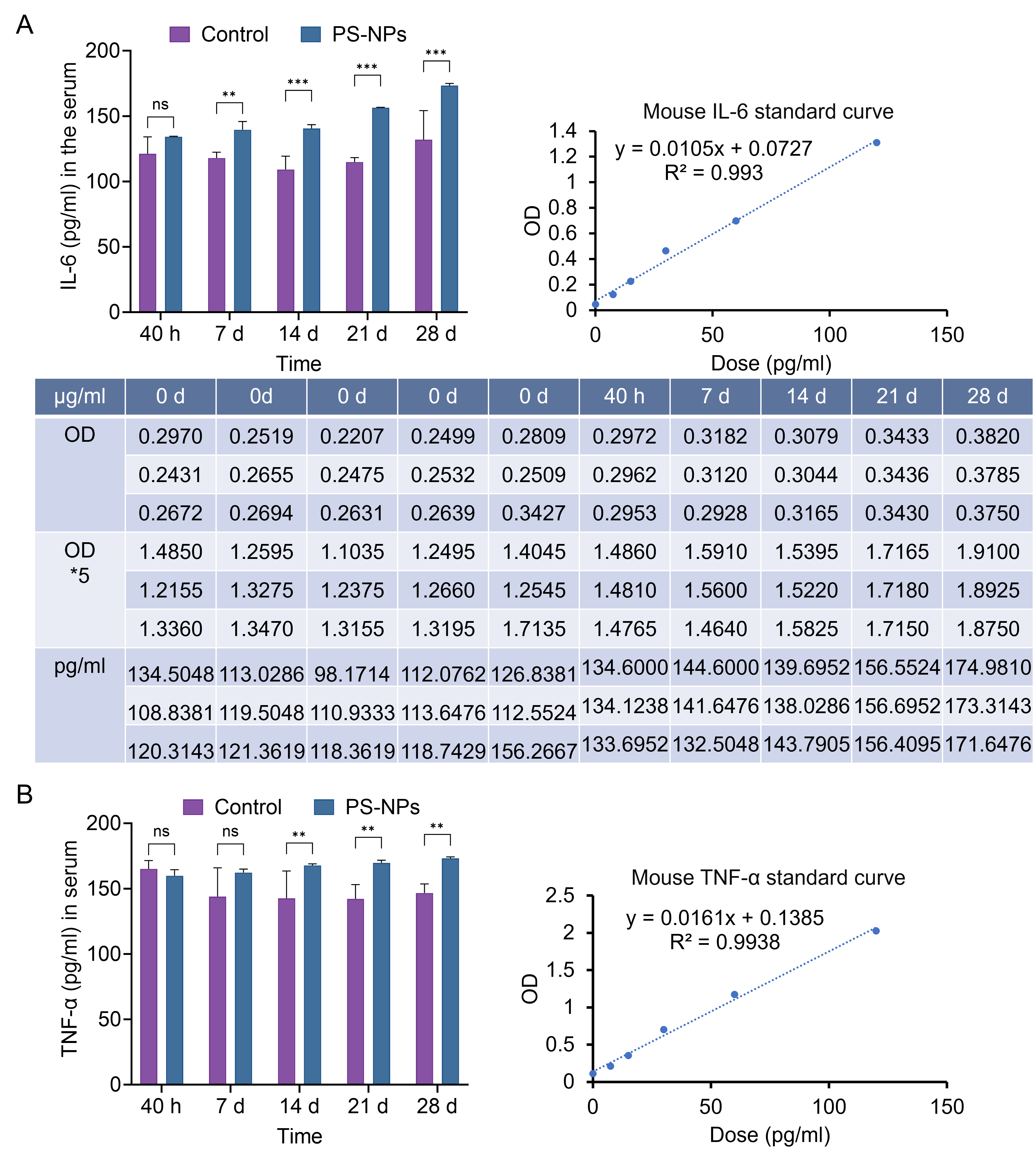


**Figure S24.** The expression levels of IL-6 (A) and TNF-α (B) in the serum of Balb/c mice treated with PS NPs. Ns is not significant, ***P* < 0.01, ****P* < 0.001 compared to the 0 μg/ml group.


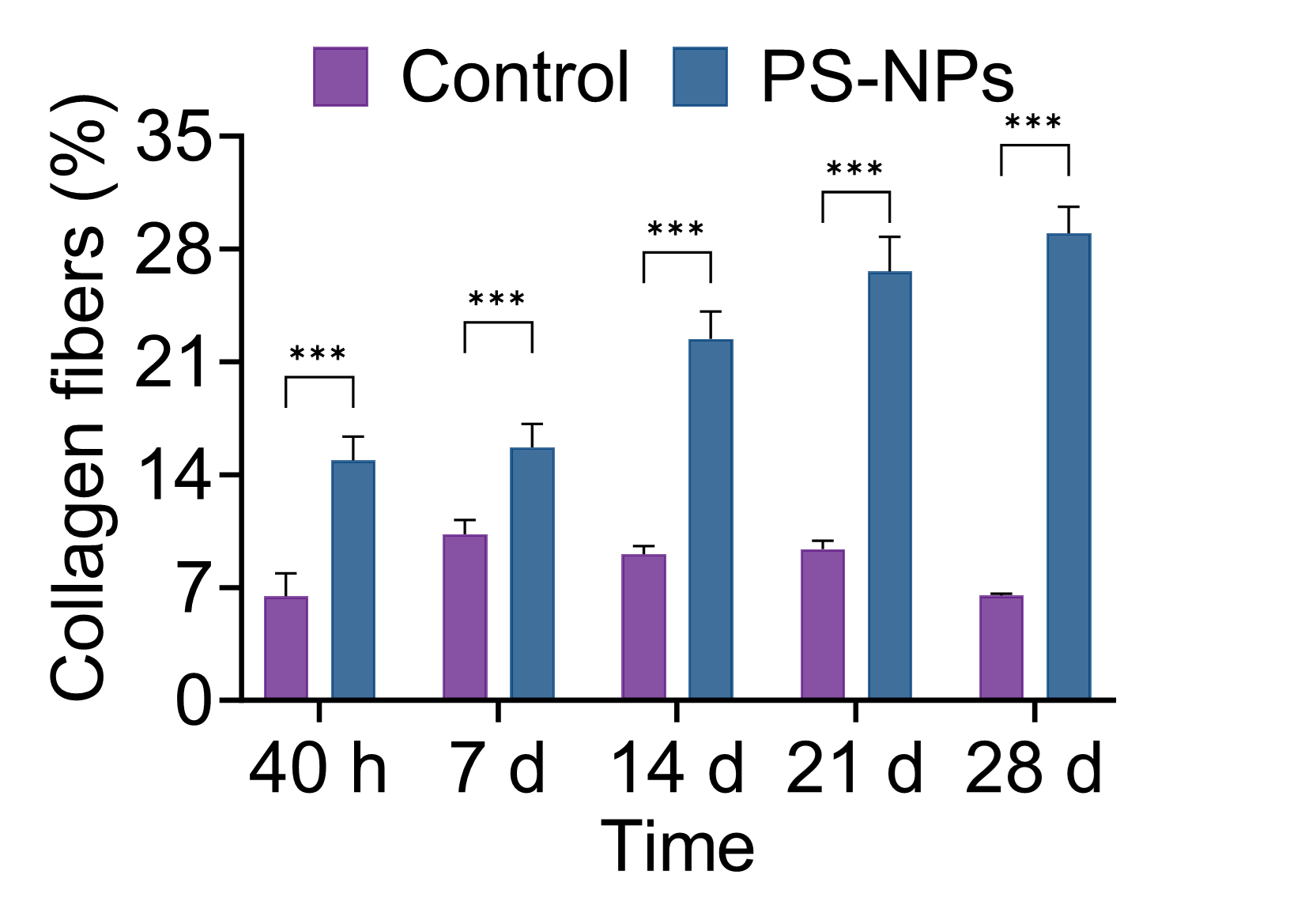


**Figure S25.** The analysis of collagen fibers based on Masson staining. ****P* < 0.001 compared to the control group.

**
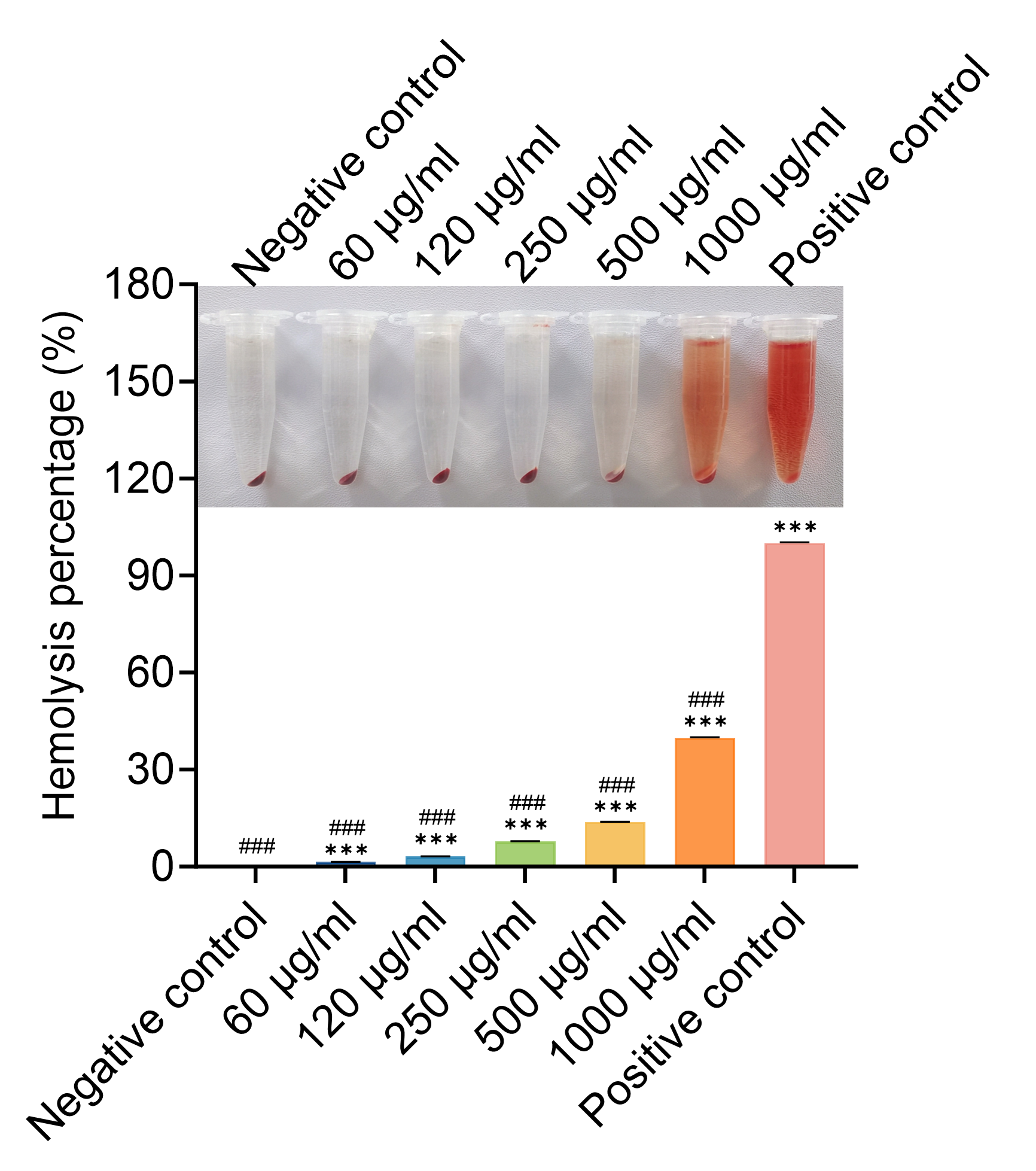
**

**Figure S26.** The hematological activity. Mouse RBCs were exposed to PS NPs at 60 - 1000 µg/ml for 3 h, and the absorbance of the released hemoglobin from cells was recorded at 541 nm. Inset photograph showing the hemoglobin appears red in the RBCs supernatant. ****P*< 0.001 compared to the negative control group. ^###^*P*< 0.001 compared to the positive control group.


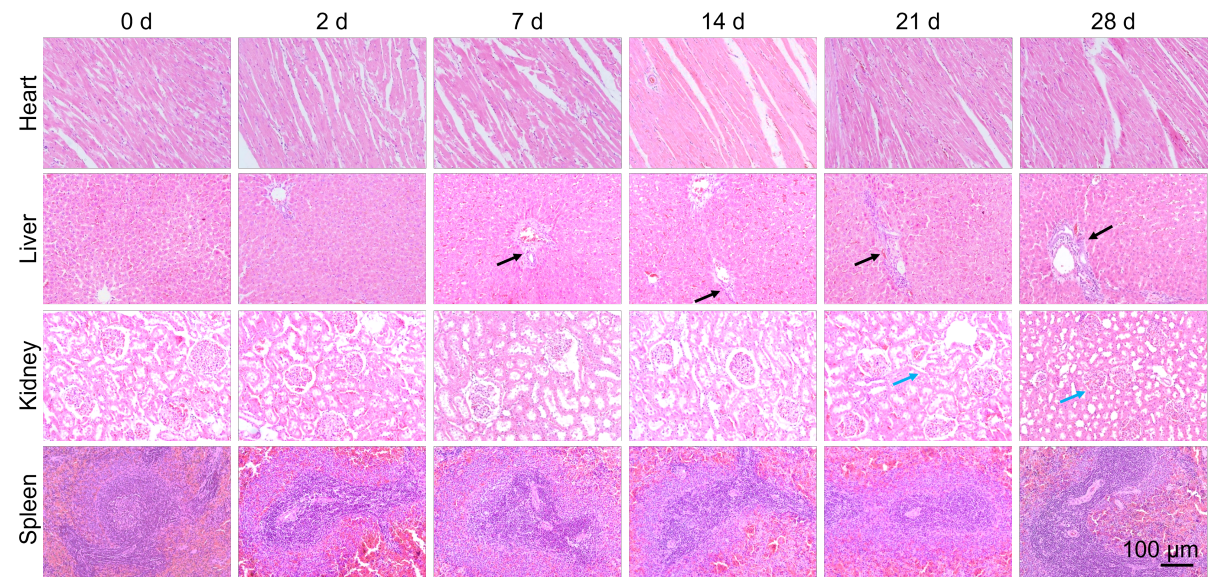
 **Figure S27.** H&E staining of the heart, liver, kidney, and spleen from Balb/c mice in each group. Black arrows indicate inflammatory infiltration; blue coloration denotes glomerular atrophy.
